# HLA‐A, ‐B, and DR Mismatch and Outcomes After Simultaneous Pancreas‐Kidney Transplantation in the Modern Era

**DOI:** 10.1111/ctr.70653

**Published:** 2026-08-24

**Authors:** Moksh Sachdeva, Samuel Tingle, Georgios Kourounis, Abdullah Malik, Emily Thompson, Steve White, Neil S. Sheerin, Colin Wilson

**Affiliations:** ^1^ Translational and Clinical Research Institute Newcastle University Newcastle Upon Tyne UK; ^2^ Institute of Transplantation Freeman Hospital Newcastle Upon Tyne UK

## Abstract

**Background:**

HLA mismatch is known to impact post‐transplant outcomes following kidney‐alone transplantation. However, the impact on kidney outcomes in the context of simultaneous pancreas‐kidney (SPK) transplantation remains uncertain. This study aimed to assess the impact of HLA mismatch on SPK outcomes.

**Method:**

Adult SPK recipients in the UNOS STAR registry (2010–2023) were analysed. Re‐transplants were excluded. Multivariable nonlinear regression models evaluated the impact of HLA mismatch on outcome.

**Results:**

In a cohort of 10,559 SPK recipients overall mismatch was frequent, with only 34 recipients (0.3%) having 0/0/0 mismatches across HLA‐A, HLA‐B, and HLA‐DR. HLA‐DR mismatch was not associated with pancreas or kidney graft survival at 5 years; two versus zero HLA‐DR mismatches aHR = 1.015 (95% CI 0.828–1.243) for pancreas graft survival, and aHR = 0.946 (0.753–1.188) for kidney graft survival. HLA‐DR mismatch was also not associated with pancreas or kidney acute rejection or 12‐month eGFR. HLA‐A and HLA‐B mismatches also showed no association with any pancreas or kidney transplant outcomes. Three‐way interaction analyses between HLA‐A, B and DR demonstrated no evidence that mismatch at any locus affected outcomes, irrespective of mismatch level at the other loci.

**Conclusion:**

In first‐time simultaneous pancreas–kidney transplantation, donor–recipient HLA mismatch was not associated with pancreas or kidney graft survival, rejection, or early renal function. These findings contrast with kidney‐alone transplantation and suggest that classical HLA mismatch effects may be attenuated in the contemporary SPK setting. Prioritising HLA‐based matching, including HLA‐DR, is therefore unlikely to improve short‐ or medium‐term post‐transplant outcomes in first‐time SPK transplantation.

## 1 Introduction

Human leukocyte antigen (HLA) mismatching between donor and recipient is a well‐established determinant of graft outcomes following kidney‐alone transplantation. Increasing degrees of HLA incompatibility are associated with higher rates of acute rejection, inferior long‐term kidney graft survival and increased alloimmune activation, despite advances in immunosuppressive therapy [1, 2]. Large registry analyses have demonstrated a stepwise reduction in kidney graft survival with increasing overall HLA mismatch burden, an effect that persisted after adjustment for centre effects and across immunosuppressive eras [3, 4]. Among the classical HLA loci, mismatching the HLA‐DR locus has been shown to exert a particularly strong influence on kidney transplant outcomes. Single‐centre studies spanning different immunosuppressive eras similarly demonstrate a strong association between HLA‐DR mismatch and inferior kidney‐alone graft outcomes, particularly in the presence of prolonged cold ischaemic times [5, 6].

However, in simultaneous pancreas‐kidney (SPK) transplantation, HLA matching has historically been deemed to be less important for the pancreas, with studies highlighting limited impact on pancreas graft survival [7]. As a result, HLA matching is often not a priority in SPK transplants [8], and recipients often go on to receive organs with multiple HLA mismatches. Despite the well‐established adverse impact of HLA mismatch in kidney‐alone transplantation, its relevance to kidney outcomes in SPK transplantation remains uncertain [9].

In SPK transplantation, kidney outcomes are linked to sustained pancreas graft function and the metabolic benefit of diabetes reversal. Registry analyses indicates that functioning pancreas grafts are associated with superior kidney survival, whereas early pancreas graft failure results in long term kidney outcomes comparable to kidney alone transplantation [10]. Long‐term observational data further suggests that sustained normoglycaemia following SPK may confer a survival advantage, indicating that metabolic factors play an important role in determining long‐term outcomes beyond immunological considerations alone [11, 12].

Contemporary SPK recipients differ substantially from kidney‐alone transplant recipients in several respects that may modify the clinical consequences of HLA incompatibility, including more intensive induction and maintenance immunosuppression, younger donor age and characteristically shorter ischaemic times. Kidney outcomes specifically in SPK recipients are influenced by multiple competing factors, including pancreas graft function, peri‐operative variables, and recipient characteristics. Within this distinct clinical and transplant context, whether HLA mismatching independently contributes to kidney graft survival remains uncertain.

This study represents the largest analysis to date specifically examining the impact of donor‐recipient HLA mismatching on kidney allograft outcomes following simultaneous pancreas–kidney transplantation. We aimed to evaluate whether HLA mismatch, which is a well‐established determinant of graft survival in kidney‐alone transplantation, exerts a similar influence on kidney graft survival in the distinct immunological context of SPK transplantation.

## 2 Methods

This population cohort study was conducted using Organ Procurement & Transplantation Network (OPTN) data obtained from the United Network for Organ Sharing Standard Transplant Analysis and Research files (UNOS STAR). Study‐specific ethical review, approval, or informed consent were not required. We included adult (aged 18 or over at the time of transplant) recipients of SPK transplants performed between January first 2010 and 30^th^ September 2023. Exclusion criteria included re‐transplants for kidney or pancreas, multiorgan transplants other than SPK, records with missing HLA‐DR mismatch data, and donors with history of diabetes or insulin dependence. Data was extracted on 30^th^ September 2024, which served as the common closure date of the study, ensuring a minimum of 1‐year post‐transplant follow up for all included patients. The primary objective of this study was to evaluate the impact of donor–recipient HLA‐DR mismatch on graft survival following simultaneous pancreas–kidney transplantation. In the UNOS STAR dataset, HLA mismatching is coded at the antigen level for the HLA‐A, HLA‐B, and HLA‐DR loci, with each locus scored as 0, 1, or 2 mismatches based on donor–recipient antigen differences. The primary outcomes assessed were kidney graft survival and pancreas graft survival. Secondary outcomes included treated acute rejection of the kidney and pancreas and renal function at one‐year post‐transplant.

Pancreas and kidney graft survival were defined using UNOS STAR registry criteria as the time from transplant until graft failure, censored at loss to follow up or 5 years. The primary outcome was recipient long‐term graft survival. UNOS STAR defines graft loss in pancreas transplantation as removal of the transplanted pancreas, recipient re‐registered for pancreas or islet transplant, recipient returned to ≥0.5 units per kilogram per day of insulin for a duration of >90 days, or recipient death [13]. Graft failure was defined as return to dialysis, re‐transplantation or death. Acute rejection outcomes represented episodes of biopsy proven or clinically treated rejection reported within the first post‐transplant year. Renal function was assessed using the estimated glomerular filtration rate (eGFR) at one year, calculated using the CKD‐EPI equation (without race adjustment) [14]. Kidney delayed graft function was defined as the requirement for dialysis in the first week post‐transplant.

## 3 Statistical Analysis

Our statistical analysis approach matches that described previously. To account for missing data, multiple imputation was performed (aregImpute; Hmisc R package in R, version 4.1.2 [R Project for Statistical Computing]) to generate 20 imputed datasets [15]. This uses predictive mean matching with bootstrap draws to build rich additive restricted cubic spline models, preserving nonlinear relationships [16]. Multiple imputation included variables listed in Table S1. Outcome variables were included as predictors in the multiple imputation model (survival outcomes as cumulative hazard and censoring indicator), to preserve associations between variables and outcome [17, 18].

To assess the association of mismatch with outcome multivariable regression modelling was performed, pooling results from all 20 imputed datasets, adjusting variance based on both within‐ and between‐imputation variation, using the fit.mult.impute function (Hmisc package in R, version 4.1.2) [15]. Adjustment for a wide range of confounders was performed. Potential confounders were selected based on previous research and clinical expertise; statistical variable selection techniques (eg, stepwise selection) were avoided [19]. Cox regression, logistic regression and linear regression were used for survival, binary (acute rejection) and continuous (eGFR) outcomes, respectively.

In these models, continuous covariates known to have a large impact on outcome or a nonlinear relationship with outcome were analysed using restricted cubic splines (RCS). This avoids assumptions of linear associations and offers greater power than splitting into arbitrary groups. For all analyses 3 knots were used, placed at the 10^th^, 50^th^ and 90^th^ percentile [15].

As the impact of HLA‐DR mismatch on outcome may be modified by the presence of other mismatches (for example, being less impactful when there are frequent A/B mismatches) we performed a three‐way interaction. This adjusted for all of the same covariates as the main models and assessed whether any potential impact of HLA‐DR mismatch on outcome was modified by the presence of HLA‐A or HLA‐B mismatches.

Kaplan‐Meier plots represent crude and non‐imputed data. All *p* values were from 2‐sided tests and results were deemed statistically significant at P < .05. All analyses were performed in R, version 4.5.1 (R Project for Statistical Computing) [20] using the following packages: rms, Hmisc, and survminer [15, 16, 21, 22]. Tables and spline plots were generated using the rmsMD package [23, 24].

## 4 Results

From 2010 to 2023, a total of 10,559 SPK recipients were included in the analysis. A CONSORT diagram (Figure S1) representing the cohort selection provides information on the number of exclusions and the final study population. Cohort demographics, stratified by HLA‐DR mismatch status are given in Table 1 (full cohort demographics for all variables, including data on missingness, is provided in Table S1). Median donor age was 23 years (IQR, 19–30), and the median recipient age was 42 years (IQR, 35–49). Median pancreas preservation time was 9.95 h (IQR, 7.50–13.0), and median kidney cold ischaemic time was 9.88 h (IQR, 7.30–13.1). Most recipients received antithymocyte globulin (ATG) as induction therapy (66.5%), followed by Alemtuzumab (14.9%) and Basiliximab, with or without ATG (8.8%) with each recipient classified according to a mutually exclusive induction category. Steroid induction was administered in 63.8% of transplants. Maintenance immunosuppression predominantly consisted of tacrolimus‐based therapy in combination with mycophenolate mofetil or mycophenolic acid (MMF/MPA), used in 93.7% of patients.

**TABLE 1:** Cohort demographic characteristics.

Characteristics	0 HLA DR mismatches (*N* = 606)	1 HLA DR mismatch (*N* = 4245)	2 HLA DR mismatches (*N* = 5708)	Overall (*N* = 10559)
**HLA‐A mismatches**				
0	72 (11.9%)	334 (7.9%)	365 (6.4%)	771 (7.3%)
1	261 (43.1%)	1896 (44.7%)	2334 (40.9%)	4491 (42.5%)
2	273 (45.0%)	2015 (47.5%)	3009 (52.7%)	5297 (50.2%)
**HLA‐B mismatches**				
0	62 (10.2%)	98 (2.3%)	43 (0.8%)	203 (1.9%)
1	197 (32.5%)	1232 (29.0%)	1261 (22.1%)	2690 (25.5%)
2	347 (57.3%)	2915 (68.7%)	4404 (77.2%)	7666 (72.6%)
**Donor age**				
Median [Q1,Q3]	23.0 [19.0,29.0]	23.0 [19.0,30.0]	23.0 [18.0,30.0]	23.0 [19.0,30.0]
**Donor Sex**				
Female	188 (31.0%)	1331 (31.4%)	1645 (28.8%)	3164 (30.0%)
Male	418 (69.0%)	2914 (68.6%)	4063 (71.2%)	7395 (70.0%)
**Donor BMI**				
Median [Q1,Q3]	23.6 [21.2,26.3]	23.7 [21.2,26.3]	23.7 [21.2,26.4]	23.7 [21.2,26.3]
**Donor Type**				
DCD donor	24 (4.0%)	135 (3.2%)	158 (2.8%)	317 (3.0%)
**Pancreas Preservation Time**				
Median [Q1,Q3]	10.0 [7.59,13.5]	9.90 [7.48,13.0]	9.98 [7.50,13.0]	9.95 [7.50,13.0]
**Kidney Cold Ischaemic Time**				
Median [Q1,Q3]	10.0 [7.50,13.8]	9.85 [7.29,13.2]	9.88 [7.30,13.0]	9.88 [7.30,13.1]
**Recipient Age**				
Median [Q1,Q3]	41.0 [35.0,48.0]	42.0 [35.0,49.0]	41.0 [35.0,49.0]	42.0 [35.0,49.0]
**Recipient Sex**				
Female	281 (46.4%)	1629 (38.4%)	2143 (37.5%)	4053 (38.4%)
Male	325 (53.6%)	2616 (61.6%)	3565 (62.5%)	6506 (61.6%)
**Recipient BMI**				
Median [Q1,Q3]	25.1 [22.5,27.8]	25.3 [22.7,28.3]	25.3 [22.9,28.1]	25.3 [22.8,28.2]
**Induction Therapy**				
ATG	408 (67.3%)	2846 (67.0%)	3769 (66.0%)	7023 (66.5%)
ATG+Basiliximab	18 (3.0%)	154 (3.6%)	205 (3.6%)	377 (3.6%)
Basiliximab without ATG	39 (6.4%)	232 (5.5%)	280 (4.9%)	551 (5.2%)
Alemtuzumab	87 (14.4%)	633 (14.9%)	852 (14.9%)	1572 (14.9%)
Other	54 (8.9%)	380 (9.0%)	602 (10.5%)	1036 (9.8%)
**Steroid Induction: yes**	391 (64.5%)	2735 (64.4%)	3608 (63.2%)	6734 (63.8%)
**Tacrolimus + MMF/MPA maintenance: yes**	576 (95.0%)	3979 (93.7%)	5342 (93.6%)	9897 (93.7%)
**CPRA Category**				
≤20	413 (68.2%)	3354 (79.0%)	4656 (81.6%)	8423 (79.8%)
>20	178 (29.4%)	767 (18.1%)	857 (15.0%)	1802 (17.1%)

*Notes:* Demographic and clinical characteristics of SPK transplant recipients according to the number of HLA‐DR mismatches.

Abbreviations: ATG, antithymocyte globulin; BMI, body mass index; DCD, donation after circulatory death; MMF/MPA, mycophenolate mofetil; SPK, simultaneous pancreas‐kidney.

**FIGURE 1:**
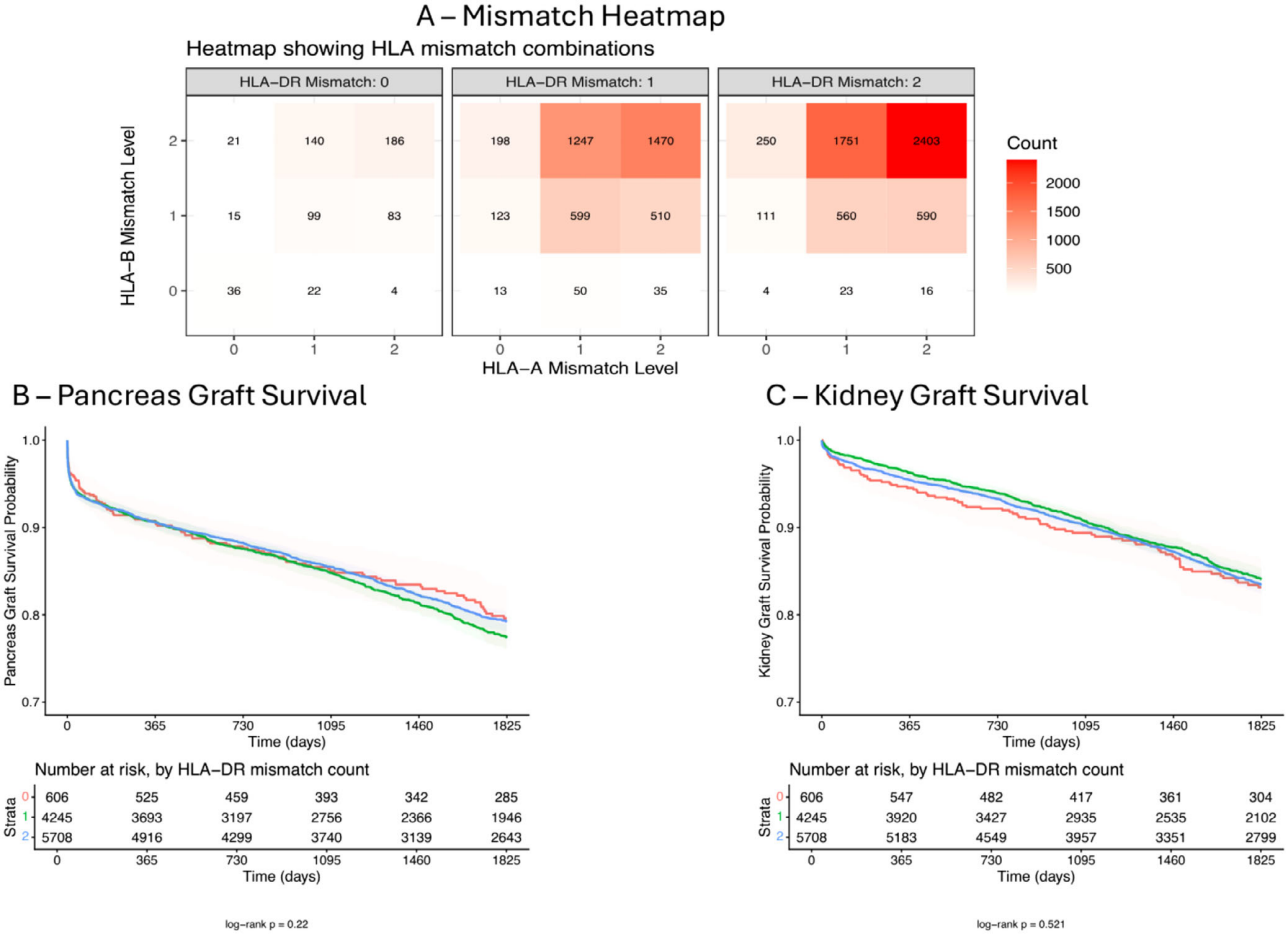
(A) HLA Mismatch Level Heatmap displaying the distribution of HLA‐A, HLA‐B and HLA‐DR mismatches among SPK transplant recipients, each recipient contributes to 1 single box. (B) Kaplan‐Meier curves showing pancreas graft survival stratified by degree of HLA‐DR mismatches (0, 1 or 2), with survival being defined according to UNOS criteria and censored at 5 years. (C) Kaplan‐Meier curves showing kidney graft survival stratified by the number of HLA‐DR mismatches (0, 1 or 2), with graft survival being defined according to UNOS criteria and censored at 5 years.

The distribution of the overall HLA mismatch burden is presented in Figure 1 (Panel A). Most recipients received organs with ≥4 mismatches, while only 3.7% had transplants with 0 or 1 total HLA mismatches and only 34 recipients displayed a complete 0/0/0 mismatch.

Kaplan‐Meier curves for pancreas and kidney graft survival, stratified by HLA‐DR mismatch, are shown in Figure 1 (Panel B and C). Survival curves for both pancreas and kidney grafts showed no evidence of separation according to the HLA‐DR mismatch level. Five‐year graft survival rates were comparable across the groups and log‐rank testing also did not demonstrate statistically significant differences.

### 4.1 Impact of HLA Mismatches on Pancreas Graft Survival

Multivariable cox regression analyses (Table 2) demonstrated that donor‐recipient HLA matching was not associated with 5‐year pancreas graft survival in SPK transplantation. Mismatch at the HLA‐DR locus showed no association with pancreas graft survival (1 mismatch: HR 1.097, 95% CI 0.895–1.344, *p* = 0.374; 2 mismatches: HR 1.015, 95% CI 0.828–1.243, *p* = 0.889). Restricted cubic spline modelling revealed the expected relationships of ischaemic times, donor age and recipient age with pancreas graft survival as shown by Figure S2.

**TABLE 2:** Multivariable model for pancreas graft survival.

Variable	Hazard ratio (95% CI)	*p* value
**HLA‐DR mismatches**		
0	Ref	—
1	1.097 (0.895 to 1.344)	0.374
2	1.015 (0.828 to 1.243)	0.889
**HLA‐A mismatches**		
0	Ref	—
1	0.921 (0.775 to 1.096)	0.354
2	0.953 (0.803 to 1.132)	0.587
**HLA‐B mismatches**		
0	Ref	—
1	0.777 (0.573 to 1.055)	0.106
2	0.793 (0.588 to 1.069)	0.128
**Pancreas preservation solution**		
UW	Ref	—
HTK	1.345 (1.162 to 1.557)	<0.001
Other	1.119 (0.987 to 1.268)	0.078
**Induction Therapy**		
ATG	Ref	—
ATG + Basiliximab	0.953 (0.745 to 1.218)	0.699
Basiliximab without ATG	1.103 (0.908 to 1.339)	0.323
Alemtuzumab	1.012 (0.889 to 1.151)	0.857
Other	1.141 (0.979 to 1.330)	0.091
**Steroids induction: yes**	0.956 (0.864 to 1.058)	0.388
**Tacrolimus + MMF/MPA maintenance: no**	1.553 (1.270 to 1.898)	<0.001
**Donor cause of death**		
Anoxia	Ref	—
CVA/stroke	1.127 (0.951 to 1.337)	0.167
Drug Overdose	1.004 (0.840 to 1.201)	0.961
Head Trauma	1.085 (0.962 to 1.225)	0.185
Other	1.029 (0.739 to 1.434)	0.864
**DCD donor**	0.865 (0.655 to 1.142)	0.306
**Recipient BMI (per unit)**	1.031 (1.019 to 1.043)	<0.001
**Recipient diabetes: Type 2**	1.133 (1.000 to 1.283)	0.050
**On dialysis: yes**	1.101 (0.990 to 1.225)	0.077
**CPRA ≤ 20**	Ref	—
**CPRA > 20**	1.005 (0.891 to 1.134)	0.929
**RCS: Pancreas Preservation Time**	RCS terms	<0.001
**RCS: BMI of donor**	RCS terms	0.110
**RCS: Age of donor**	RCS terms	<0.001
**RCS: Treatment year**	RCS terms	0.207
**RCS: Age of recipient**	RCS terms	<0.001

*Notes:* Cox regression model evaluating the association between donor, recipient, and transplant characteristics and pancreas graft survival, adjusted for HLA mismatches and clinical covariates. Restricted cubic spline terms were included for pancreas preservation time, donor BMI, donor age, treatment year, and recipient age; spline plots are shown in Figure S2.

Abbreviations: ATG, antithymocyte globulin; CPRA; calculated panel reactive antibody; CVA, cerebrovascular accident; HTK, histidine–tryptophan–ketoglutarate; MMF/MPA, mycophenolate mofetil; RCS, restricted cubic spline; UW, University of Wisconsin solution.

Additional analyses, adjusting for all factors in Table 2, and including a three‐way interaction term for HLA‐A, HLA‐B and HLA‐DR mismatches demonstrated no significant interaction effects, irrespective of mismatch at the other loci. These findings are further illustrated in Figure S3 which demonstrates no separation between mismatch groups.

### 4.2 Impact of HLA Mismatches on Pancreas Acute Rejection

As presented in Table S2 (cubic splines in Figure S4), donor‐recipient HLA mismatching was not associated with the risk of pancreas acute rejection within the first post‐transplant year. Mismatch at the HLA‐DR locus was not associated with pancreas acute rejection at one year (1 mismatch: OR 1.186 CI 0.847–1.660, *p* = 0.321; 2 mismatches: OR 1.246, 95% CI 0.899–1.728, *p* = 0.186). Similarly, HLA‐A and HLA‐B mismatches showed no significant association with pancreas rejection (HLA‐A: *p* = 0.390 and *p* = 0.886; HLA‐B: *p* = 0.484 and *p* = 0.713), further indicating that overall, HLA mismatching did not influence rejection risk in SPK recipients. Spline analysis revealed significant non‐linear associations for donor age and transplant year; younger donor age was associated with a lower risk of rejection, with rejection risk also declining in more recent transplant times. Induction regimen was a major determinant of pancreas rejection risk, with ATG being used as the reference group. Compared to ATG, recipients receiving Basiliximab without ATG (adjusted OR 2.031, 95% CI 1.460–2.824, *p* < 0.001), Alemtuzumab (adjusted OR 1.716, 95% CI 1.409–2.090, p < 0.001) had significantly higher adjusted odds of pancreas rejection (Table S2). Steroid induction was also independently associated with increased rejection compared with steroid free regimens (OR 1.217, 95% CI 1.011–1.464, *p* = 0.038). Higher recipient BMI also predicted rejection (OR per unit 1.025, 95% CI 1.006–1.044, *p* = 0.009). Restricted cubic spline modelling demonstrated non‐linear associations, with risk of rejection being lower in more recent years (Figure S4).

### 4.3 Impact of HLA Mismatches on Kidney Graft Survival

Multivariable cox regression analyses (Table 3) demonstrated that donor‐recipient HLA matching was not associated with 5‐year kidney graft survival following SPK transplantation, and further displayed through cubic spline modelling in Figure S5. HLA‐DR mismatch was not associated with 5‐year kidney graft survival (1 mismatch, HR 0.901, 95% CI 0.715–1.134, *p* = 0.374; 2 mismatches: HR 0.946, 95% CI 0.753–1.188, *p* = 0.630). Consistent with this, HLA‐A and HLA‐B mismatches also showed no significant association with graft survival when comparing one mismatch with zero mismatches at either locus (HLA‐A: *p* = 0.526 and *p* = 0.785; HLA‐B: *p* = 0.302 and *p* = 0.574), indicating that overall HLA mismatching exerted minimal influence on kidney outcomes in the SPK setting (Figure S6). Rather, predictors of graft loss included recipient type 2 diabetes compared to type 1 diabetes (HR 1.200, 95% CI 1.035–1.391, *p* = 0.016), dialysis at the time of transplant versus not being on dialysis (HR 1.204, 95% CI 1.058–1.370, *p* = 0.005), and a lack of Tacrolimus+MMF/MPA maintenance therapy compared with Tacrolimus+MMF/MPA (HR 1.758, 95% CI 1.405–2.202, *p* < 0.001). Restricted cubic spline modelling demonstrated significant non‐linear associations between graft outcomes and cold ischaemic time, donor age, recipient age, and transplant year, with higher risk observed with longer ischaemic times and older donor and recipient age, and improved outcomes in more recent transplant eras.

**TABLE 3:** Multivariable model for kidney graft survival.

Variable	Hazard ratio (95% CI)	*p* value
**HLA‐DR mismatches**		
0	Ref	—
1	0.901 (0.715 to 1.134)	0.374
2	0.946 (0.753 to 1.188)	0.630
**HLA‐A mismatches**		
0	Ref	—
1	0.935 (0.760 to 1.150)	0.526
2	0.972 (0.792 to 1.193)	0.785
**HLA‐B mismatches**		
0	Ref	—
1	0.821 (0.564 to 1.194)	0.302
2	0.900 (0.623 to 1.300)	0.574
**Induction Therapy**		
ATG	Ref	—
ATG + Basiliximab	1.167 (0.895 to 1.521)	0.255
Basiliximab without ATG	0.912 (0.710 to 1.171)	0.469
Alemtuzumab	1.021 (0.877 to 1.188)	0.787
Other	1.137 (0.950 to 1.362)	0.162
**Steroids induction: yes**	1.058 (0.936 to 1.196)	0.364
**Tacrolimus + MMF/MPA maintenance: no**	1.758 (1.405 to 2.202)	<0.001
**Donor cause of death**		
Anoxia	Ref	—
CVA/stroke	0.905 (0.733 to 1.118)	0.355
Drug Overdose	1.064 (0.866 to 1.306)	0.557
Head Trauma	1.008 (0.876 to 1.160)	0.914
Other	1.168 (0.813 to 1.679)	0.401
**DCD donor**	0.934 (0.679 to 1.284)	0.673
**Recipient BMI (per unit)**	1.008 (0.994 to 1.022)	0.274
**Recipient diabetes: Type 2**	1.200 (1.035 to 1.391)	0.016
**On dialysis: yes**	1.204 (1.058 to 1.370)	0.005
**CPRA ≤ 20**	Ref	—
**CPRA > 20**	1.072 (0.932 to 1.233)	0.329
**RCS: Kidney Cold Ischaemic Time**	RCS terms	0.015
**RCS: BMI of donor**	RCS terms	0.655
**RCS: Age of donor**	RCS terms	<0.001
**RCS: Treatment year**	RCS terms	0.009
**RCS: Age of recipient**	RCS **terms**	<0.001
**RCS: Donor peak creatinine**	RCS terms	0.353

*Notes:* Cox regression model evaluating the association between donor, recipient, and transplant characteristics and kidney graft survival, adjusted for HLA mismatches and clinical covariates. Restricted cubic spline terms were included for cold ischaemic time, donor BMI, donor age, treatment year, recipient age, and donor peak creatinine; spline plots are shown in Figure S5.

Abbreviations: ATG, antithymocyte globulin; CPRA, calculated panel reactive antibody; CVA, cerebrovascular accident; MMF/MPA, mycophenolate mofetil; RCS, restricted cubic spline.

### 4.4 Impact of HLA Mismatches on Kidney Acute Rejection and Renal Function

As presented in Table S3 (splines presented in (Figure S7), donor‐recipient HLA mismatching was not associated with the risk of kidney acute rejection within the first post‐transplant year. Mismatch at the HLA‐DR locus was not associated with kidney acute rejection within the first year (1 mismatch: OR 0.853, 95% CI 0.621–1.172, *p* = 0.326; 2 mismatches: OR 0.979, 95% CI 0.715–1.341, *p* = 0.897). HLA–A and HLA‐B mismatches also showed no association with rejection (HLA‐A: *p* = 0.581 and *p* = 0.756; HLA‐B: *p* = 0.204 and *p* = 0.376), highlighting that overall, HLA mismatching did not influence rejection risk in SPK transplantation. Independent predictors of increased rejection risk included Alemtuzumab induction compared with ATG (OR 1.274, 95% CI 1.046–1.551, *p* = 0.016), absence of Tacrolimus+MMF/MPA maintenance (OR 1.451, 95% CI 1.034–2.037, *p* = 0.032), greater recipient BMI (OR per unit 1.036, 95% CI 1.017–1.056, *p* < 0.001). Restricted cubic spline analysis showed non‐linear patterns, with greater rejection risk observed at longer cold ischaemic times, increasing recipient age, and in earlier transplant eras; all spline terms for these variables were statistically significant (*p* < 0.05) (Figure S7).

Consistent with these results, donor‐recipient HLA mismatching was not associated with renal allograft function at one‐year post‐transplant. Mismatch at the HLA‐DR locus did not influence adjusted 1‐year eGFR (1 mismatch: +0.637 mL/min/1.73m^2^, 95% CI −1.425 to 2.699, *p* = 0.545; 2 mismatches: −0.511, 95% CI −2.560 to 1.539, *p* = 0.625). Determinants of renal function included donor head trauma as cause of death (+2.010, 95% CI 0.785–3.234, *p* = 0.001), and higher recipient BMI (per unit: −0.707, 95% CI −0.828 to −0.586, *p* < 0.001) (Table S4). Restricted cubic spline analyses further demonstrated non‐linear associations with poorer function at older donor and recipient ages, higher donor BMI, and prolonged cold ischaemic times (Figure S8). As expected, HLA mismatch was also not associated with kidney delayed graft function (Figure S9 and Table S5).

### 4.5 Sensitivity Analyses Including Hierarchical Models

Sensitivity analyses provided further evidence for the stability of the method—hierarchical frailty models incorporating centre‐level effects showed substantial variation in outcomes across transplant centres; however, HLA mismatch itself remained unassociated with either pancreas or kidney graft survival. Additional modelling including three‐way interaction terms for HLA‐A, HLA‐B and HLA‐DR mismatches demonstrated no significant interaction effects, with no evidence that mismatch at any individual locus influenced outcomes, irrespective of mismatch at other loci. Finally, landmark analyses restricted to pancreas and kidney grafts surviving beyond 30 days, thereby excluding early graft losses likely to predominantly reflect technical failure, produced findings consistent with the primary graft survival models in Tables 2 and 3.

## 5 Discussion

In this national cohort of 10,559 first‐time simultaneous pancreas–kidney (SPK) transplants, HLA‐A, B or DR mismatch was not associated with pancreas or kidney graft survival, acute rejection, or one‐year renal function. These findings were consistent across multivariable models, centre‐level sensitivity analyses, and three‐way interactions between HLA‐A, HLA‐B, and HLA‐DR mismatches. This represents the most extensive evaluation to date of HLA mismatch in SPK transplantation and contrasts with the well‐established adverse impact of HLA mismatch, especially HLA‐DR mismatch, in kidney‐alone transplantation [1].

This apparent divergence from kidney‐alone transplantation is likely explained by transplant related factors that modulate early alloimmune activation. In particular, cold ischaemic time (CIT) plays a key role in shaping the clinical relevance of HLA mismatching. Prior registry analyses have demonstrated that the adverse impact of HLA mismatching on graft survival in kidney transplantation is most evident in the setting of prolonged cold ischaemic time [25]. Prolonged ischaemia promotes endothelial injury and augments antigen presentation, thereby amplifying alloimmune responses [26]. Consequently, shorter preservation times in SPK transplantation, together with the predominance of young donors, may substantially attenuate early alloimmune activation and reduce the clinical impact of donor‐recipient HLA incompatibility. Support for this interpretation is provided by evidence from living donor kidney transplantation, in which donors are typically younger and cold ischaemic times are minimal, and where HLA mismatching has been shown to exert a markedly weaker influence on graft outcomes compared with deceased donor transplantation [27, 28]. The parallels between SPK and living donor kidney transplantation are particularly telling in this regard, as both settings combine favourable donor characteristics with minimised preservation times that together appear to minimise the immunological penalty of HLA disparity. In a large registry analysis from the UK transplant registry, five‐year allograft survival in children receiving poor HLA‐matched living donor kidneys was not inferior to that of recipients of well‐matched deceased donor grafts, leading to questioning of the rationale for awaiting a better‐matched deceased donor organ [29]. Comparable findings have been reported in adult cohorts, in which recipients of high mismatch living donor transplants achieved graft survival superior to that of low‐mismatch deceased donor recipients, reinforcing the notion that donor quality and ischaemic conditions may outweigh HLA compatibility in determining early and immediate‐term outcomes [30]. This pattern mirrors that observed in the present SPK cohort, in which the combination of young donors, short cold ischaemic times and potent contemporary immunosuppression appears to make the contribution of HLA mismatch to graft outcome negligible.

In addition to these donor and preservation related factors, contemporary immunosuppressive practice in SPK transplantation is likely to further mitigate the clinical consequences of HLA mismatch. In our cohort (Table 1), two‐thirds of the patients received antithymocyte globulin induction and over 90% were maintained on tacrolimus with mycophenolate, regimens that strongly suppress T‐cell mediated immune responses. Within this contemporary immunosuppressive context, any damaging effects of HLA mismatch on both pancreas and kidney grafts may have been mitigated.

Importantly, the attenuation of classical HLA mismatch effects observed in the present SPK cohort is unlikely to reflect a pancreas‐mediated immunoprotective effect analogous to that seen in simultaneous liver kidney transplantation. Unlike the liver, the pancreas has no established role in antibody sequestration or systemic immunoregulation, Rather the absence of an association between HLA mismatch and graft outcomes in SPK transplantation is more plausibly explained by contemporary transplant practice characteristics, including younger donor age, short cold ischaemic times and the widespread use of potent induction and maintenance immunosuppression, all of which are known to limit early alloimmune activation.

Within this context, SPK transplantation is also characterised by heterogeneous, organ‐specific alloimmune responses. Concurrent biopsy studies demonstrate that rejection in SPK recipients is frequently organ‐specific, with more severe rejection often observed in the pancreas when both grafts are involved. In a cohort of SPK recipients undergoing biopsy for graft dysfunction, Uva et al. analysed 101 kidney and/or pancreas biopsies from 70 patients and found pancreas‐only and kidney‐only rejection to be common, with higher rejection grades typically involving the pancreas [31]. However in the present study, donor‐recipient HLA mismatching was not associated with pancreas acute rejection or graft survival, indicating that variability in organ‐specific immune injury does not translate into clinically meaningful HLA‐dependent effects on graft outcomes under contemporary immunosuppressive practice.

Extensive evidence from kidney‐alone transplantation demonstrates that HLA‐DR mismatching is associated with higher rejection rates and reduced long‐term graft survival, underpinning its inclusion in kidney allocation algorithms [1].Against this established background, the absence of an association with outcome in this cohort was not confined to HLA‐DR mismatch. Mismatching at the HLA‐A and HLA‐B loci was also not associated with pancreas or kidney graft survival, acute rejection, or one‐year renal function. This contrasts with kidney‐alone transplantation, where HLA‐A and HLA‐B mismatches have historically been associated with increased rejection risk and inferior graft survival, albeit with weaker and less consistent effects than HLA‐DR mismatch [32]. Large registry analyses from the Collaborative Transplant Study have demonstrated incremental reductions in kidney graft survival with increasing HLA‐A and HLA‐B mismatches, particularly in earlier immunosuppressive eras and in the presence of prolonged cold ischaemic times [4]. More contemporary studies suggest that while the impact of HLA‐A and HLA‐B mismatching has diminished under modern immunosuppression, residual effects may persist in kidney‐alone transplantation [1].

Studies in SPK transplantation have shown differing results, with many showing no significant effect [7, 33], with few showing increases in rejection [8, 34]. These observations are supported by Garg et al., who showed that although greater HLA mismatching between recipients and third‐party donor vascular allografts used in pancreas transplantation was associated with an increased risk of de novo donor‐specific antibody formation, this did not translate into inferior kidney or pancreas graft outcomes under contemporary immunosuppression [33]. Similarly, Rudolph et al. reported no association between HLA matching and graft or patient survival across pancreas transplant categories in a large single‐centre cohort, with donor factors such as age and preservation time exerting greater influence on outcomes [7]. These results suggest that the adverse effects of HLA‐DR mismatch observed in kidney‐alone transplantation may be attenuated in the SPK setting.

Earlier work often included heterogenous immunosuppressive regimens and longer preservation times, factors that may have influenced graft outcomes and contributed to conflicting results regarding the relevance of HLA compatibility [4]. In contrast, recipients in the present cohort were managed predominantly with contemporary immunosuppression, characterised by widespread use of T‐cell–depleting induction and tacrolimus‐based maintenance in combination with mycophenolate. Consistent with this, absence of tacrolimus plus MMF/MPA maintenance was independently associated with inferior pancreas and kidney graft outcomes, whereas HLA mismatch showed no association with graft survival or rejection. These findings suggest that previously reported associations between HLA mismatch and outcome in SPK transplantation may reflect insufficient suppression of alloimmune responses under earlier immunosuppressive protocols rather than a direct biological effect of HLA locus disparity itself.

An important consideration when interpreting these findings is the duration of follow‐up. While kidney allografts frequently function for more than a decade, the present analysis was limited to five‐year outcomes, raising the possibility that late graft failures related to HLA‐DR mismatch may not yet be apparent. However, one‐year estimated glomerular filtration rate has been shown to be a strong and independent predictor of long‐term graft survival, regardless of donor, recipient, or early post‐transplant factors, with lower 12‐month eGFR associated with a substantially increased risk of subsequent graft loss [7, 35]. In this context, the absence of any association between HLA‐DR mismatch and graft survival, rejection, or one‐year eGFR provides meaningful evidence that HLA‐DR mismatch does not exert a substantial early or intermediate‐term impact on SPK outcomes under contemporary practice. We excluded pancreas retransplants here, as they represent a unique cohort. However, dedicated study of the retransplant population is certainly warranted.

Although HLA‐DR mismatch did not affect short‐ or medium‐term graft outcomes in this cohort, its relevance to long‐term sensitisation and future re‐transplantation remains uncertain but clinically important. SPK recipients are typically younger and may require subsequent kidney transplantation later in life. Greater HLA mismatching has been associated with increased risk of donor‐specific and broadly reactive antibody formation, which can complicate future organ allocation and prolong waiting times [36]. As *de novo* donor‐specific antibody data are not recorded in the UNOS STAR registry, the impact of HLA‐DR mismatch on longitudinal sensitisation could not be assessed in this study. We also lacked granular data on post‐operative complications and readmissions. Additional limitations include the small proportion of recipients that had complete 0/0/0 HLA matches across the A, B and DR loci. As this group of complete HLA match is small, it remains uncertain whether achieving a complete A, B and DR match is beneficial. Data on HLA‐DQ and HLA‐DP mismatching and post‐transplant immunologic monitoring were unavailable, and the analysis was restricted to first‐time SPK recipients, limiting generalisability to pancreas alone or other multi‐organ transplant combinations.

## 6 Conclusion

In this large national cohort of first‐time simultaneous pancreas–kidney transplants, donor–recipient HLA mismatching was not associated with pancreas or kidney graft survival, acute rejection, or early renal function. These findings contrast with established evidence from kidney‐alone transplantation and suggest that the immunological relevance of classical HLA mismatching, including HLA‐DR, may be attenuated in the contemporary SPK setting. Short cold ischaemic times, intensive modern immunosuppression, and the unique immunological dynamics of combined organ transplantation may collectively mitigate the clinical impact of HLA incompatibility. Together, the data indicates that prioritising HLA‐based matching in current SPK allocation policies is unlikely to yield meaningful improvements in post‐transplant outcomes for first‐time SPK recipients.

## Conflicts of Interest

The authors declare no conflicts of interest.

## Funding

Samuel J. Tingle was funded for this work via a Medical Research Council Clinical Research Training Fellowship (MRC/Y000676/1), which was part funded by Kidney Research UK. This study is funded by the National Institute for Health and Care Research (NIHR) Blood and Transplant Research Unit in Organ Donation and Transplantation (NIHR203332), a partnership between NHS Blood and Transplant, University of Cambridge and Newcastle University. The views expressed are those of the author(s) and not necessarily those of the NIHR, NHS Blood and Transplant or the Department of Health and Social Care.

## Supporting information




**Supporting information**: ctr70653‐sup‐0001‐SuppMat.pdf

## Data Availability

The data used in the analysis are available from OPTN/UNOS. Data can be requested from OPTN/UNOS through the following website: https://optn.transplant.hrsa.gov/data/view‐data‐reports/request‐data/.
