# Supplementary material for: HLA‐A, ‐B, and DR Mismatch and Outcomes After Simultaneous Pancreas‐Kidney Transplantation in the Modern Era
Source: Clin Transplant. 2026 Aug 24;40(8):e70653. doi: 10.1111/ctr.70653 (PMC13503977; doi:10.1111/ctr.70653)
Supplement: Supplementary file 1 [file CTR-40-e70653-s001.pdf]

Supplementary Figures

Supplementary Figure 1: CONSORT diagram of cohort selection. The figure outlines the inclusion and exclusion criteria for the study population of simultaneous pancreas-kidney (SPK) transplants.

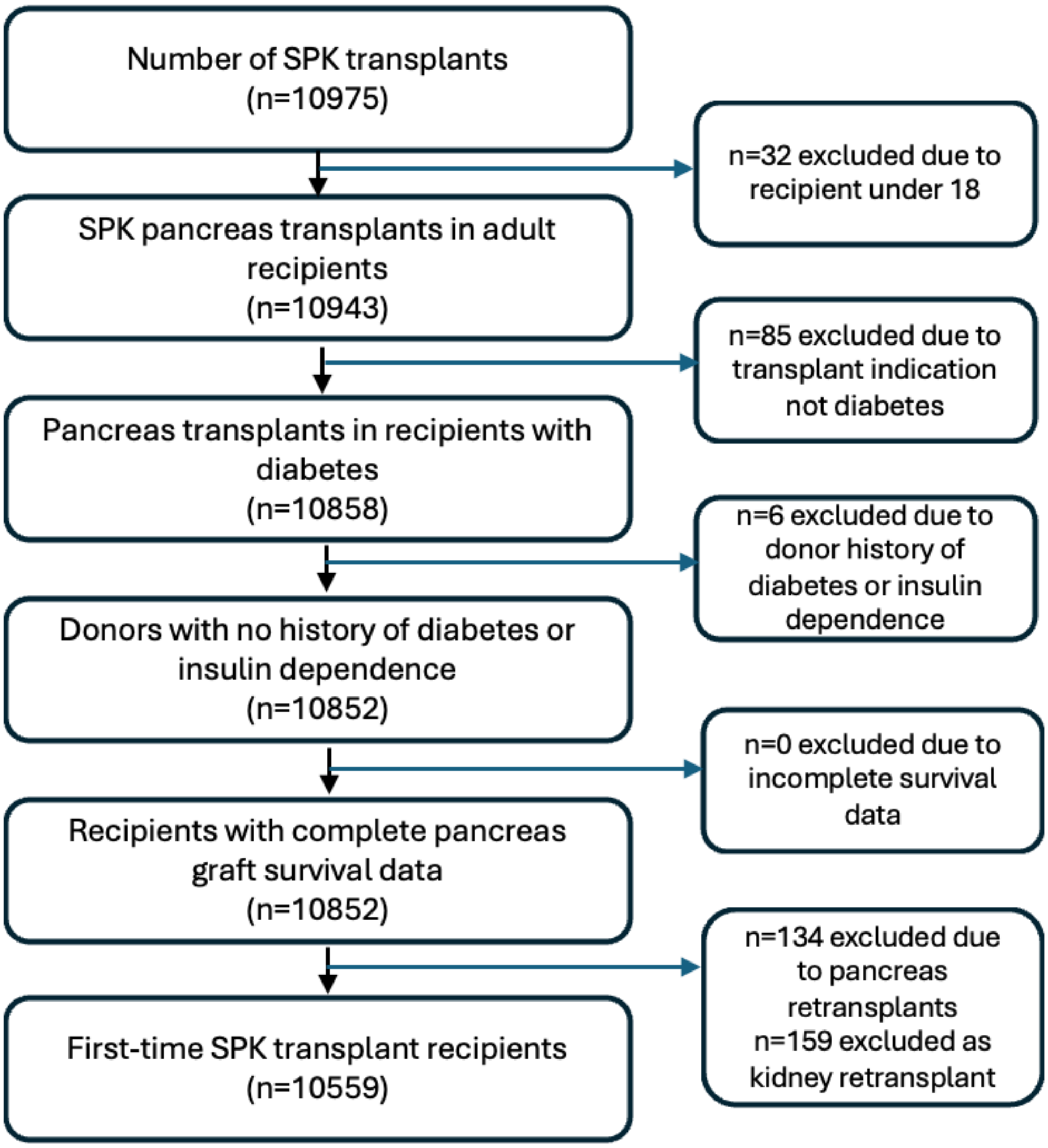

Supplementary Figure 2: Restricted cubic spline model for pancreas graft survival. Restricted cubic spline plots showing the associations of donor, recipient, and transplant characteristics with pancreas graft survival, adjusted for all co-variates in Table 2

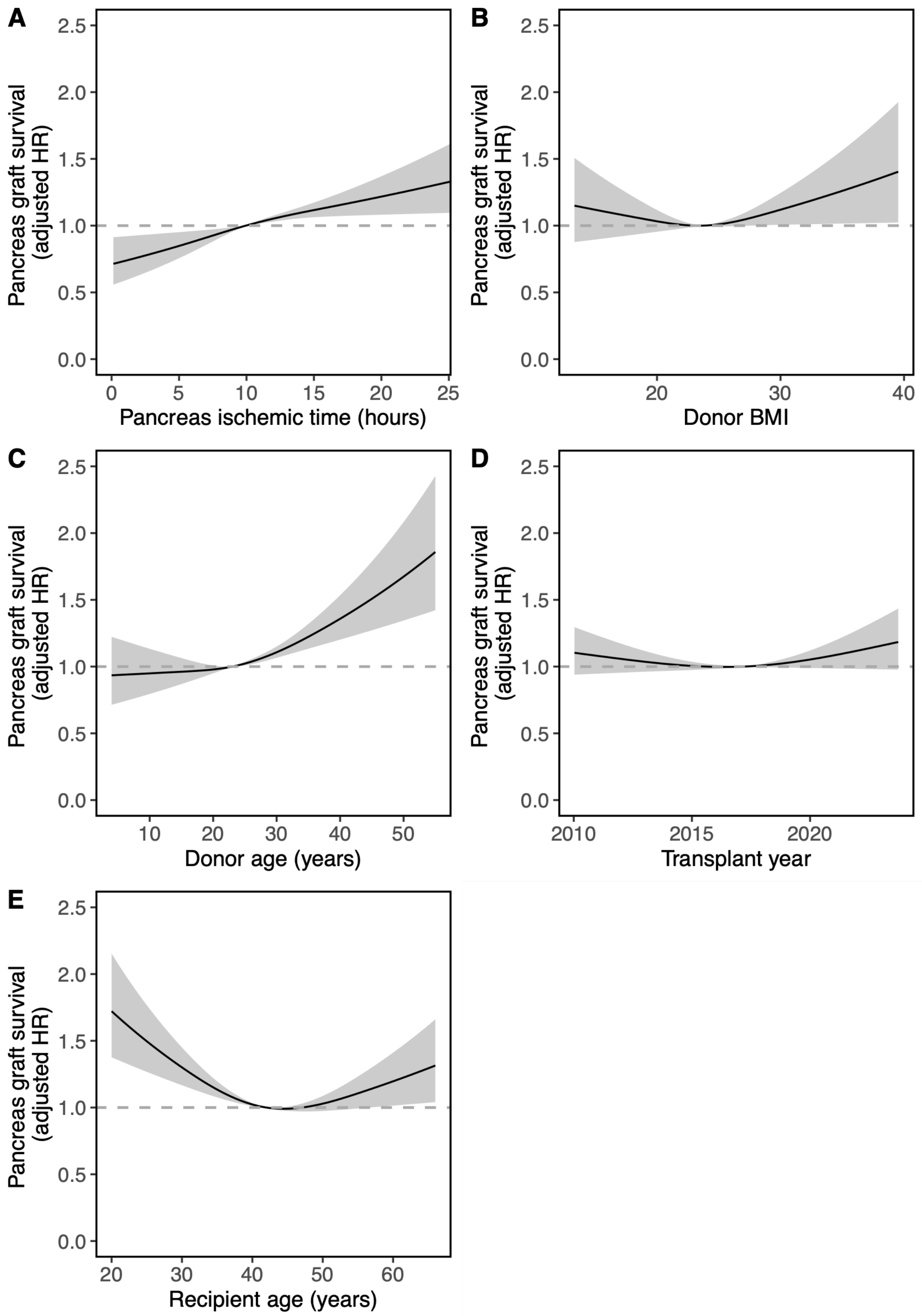

Supplementary Figure 3: Kaplan–Meier pancreas graft survival by HLA mismatch. Panel A shows the degree of HLA A mismatch, Panel B shows the degree of HLA-B mismatch, Panel C shows the overall degree of HLA mismatch

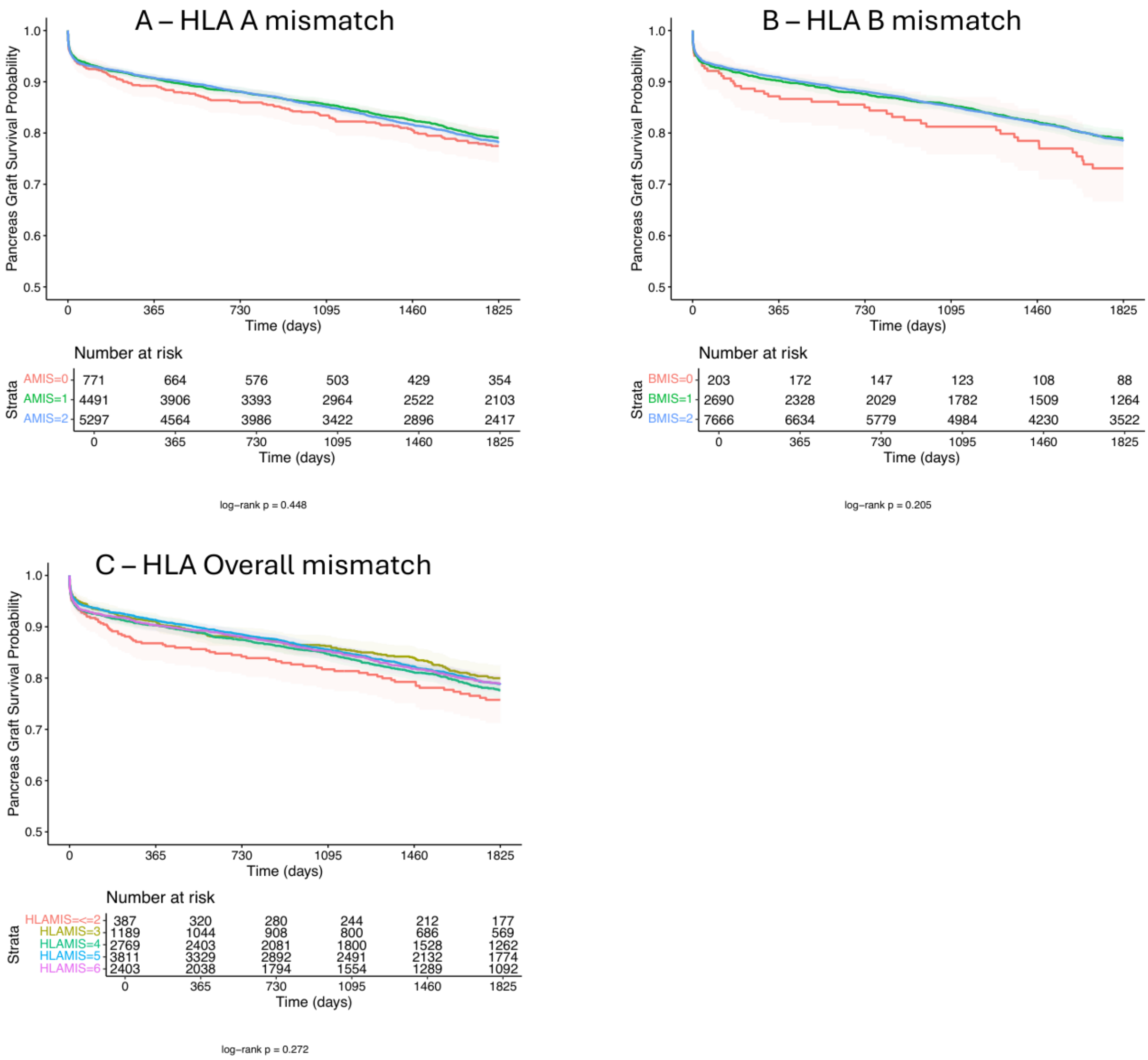

Supplementary Figure 4: Restricted cubic spline model for pancreas acute rejection. Restricted cubic spline plots showing associations of donor, recipient, and transplant characteristics with the risk of pancreas acute rejection at 1 year, adjusted for all covariates in Supplementary Table 2

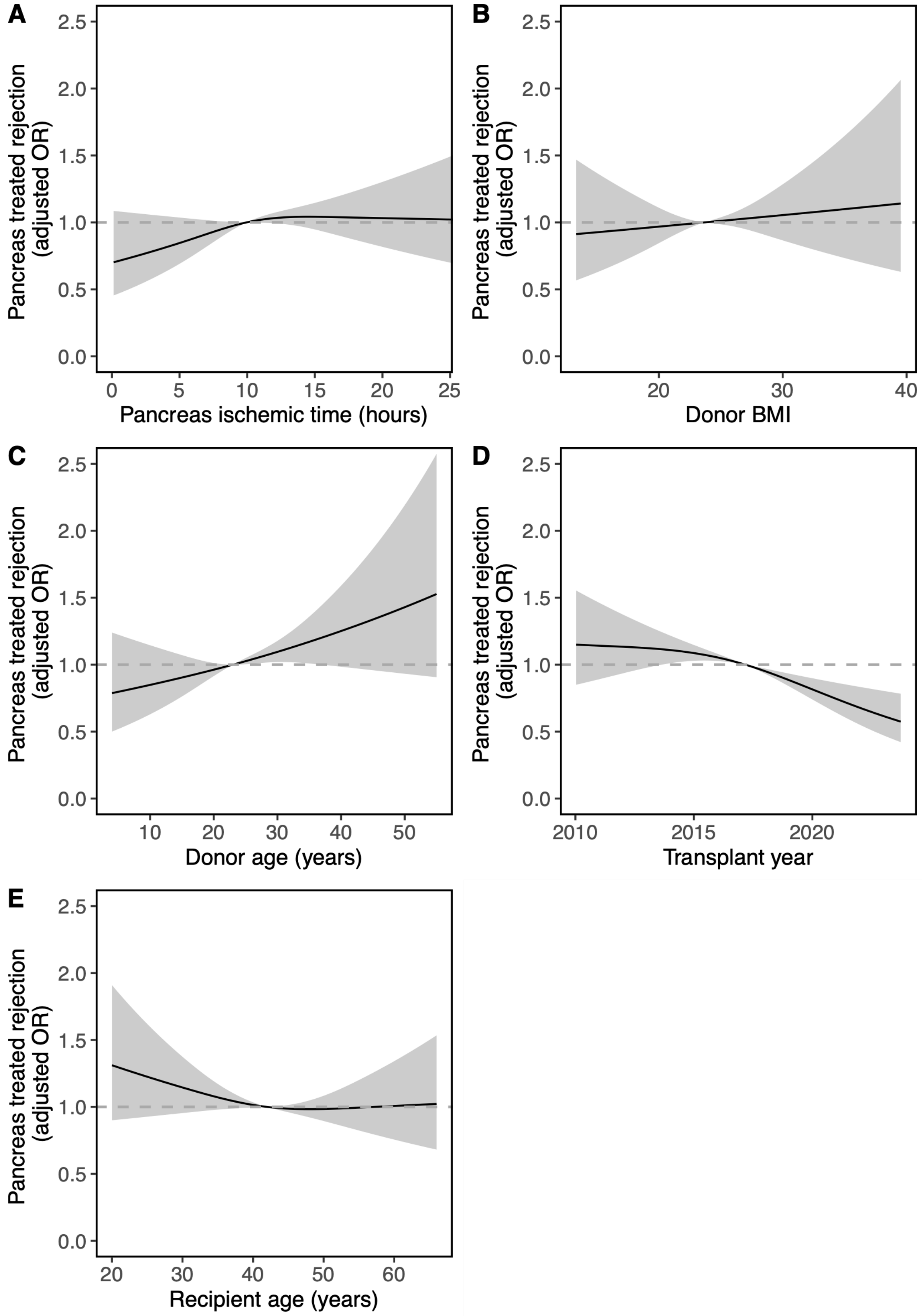

Supplementary Figure 5: Restricted cubic spline model for kidney graft survival. Restricted cubic spline plots demonstrating relationships between key transplant variables and kidney graft survival, adjusted for all covariates in Table 3

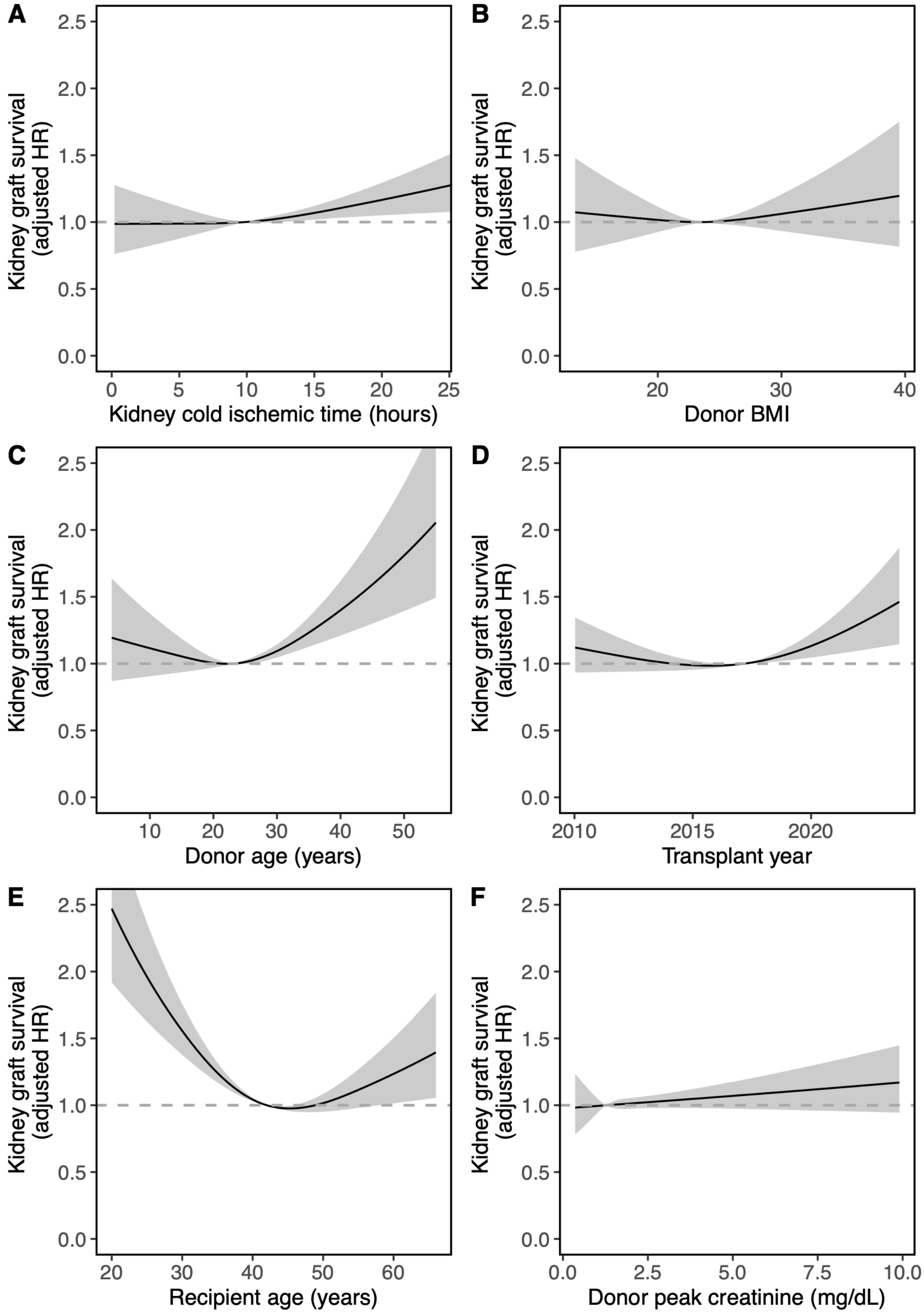

Supplementary Figure 6: Kaplan–Meier kidney graft survival by HLA mismatch. Panel A shows the degree of HLA A mismatch, Panel B shows the degree of HLA-B mismatch, Panel C shows the overall degree of HLA mismatch

A – HLA A mismatch

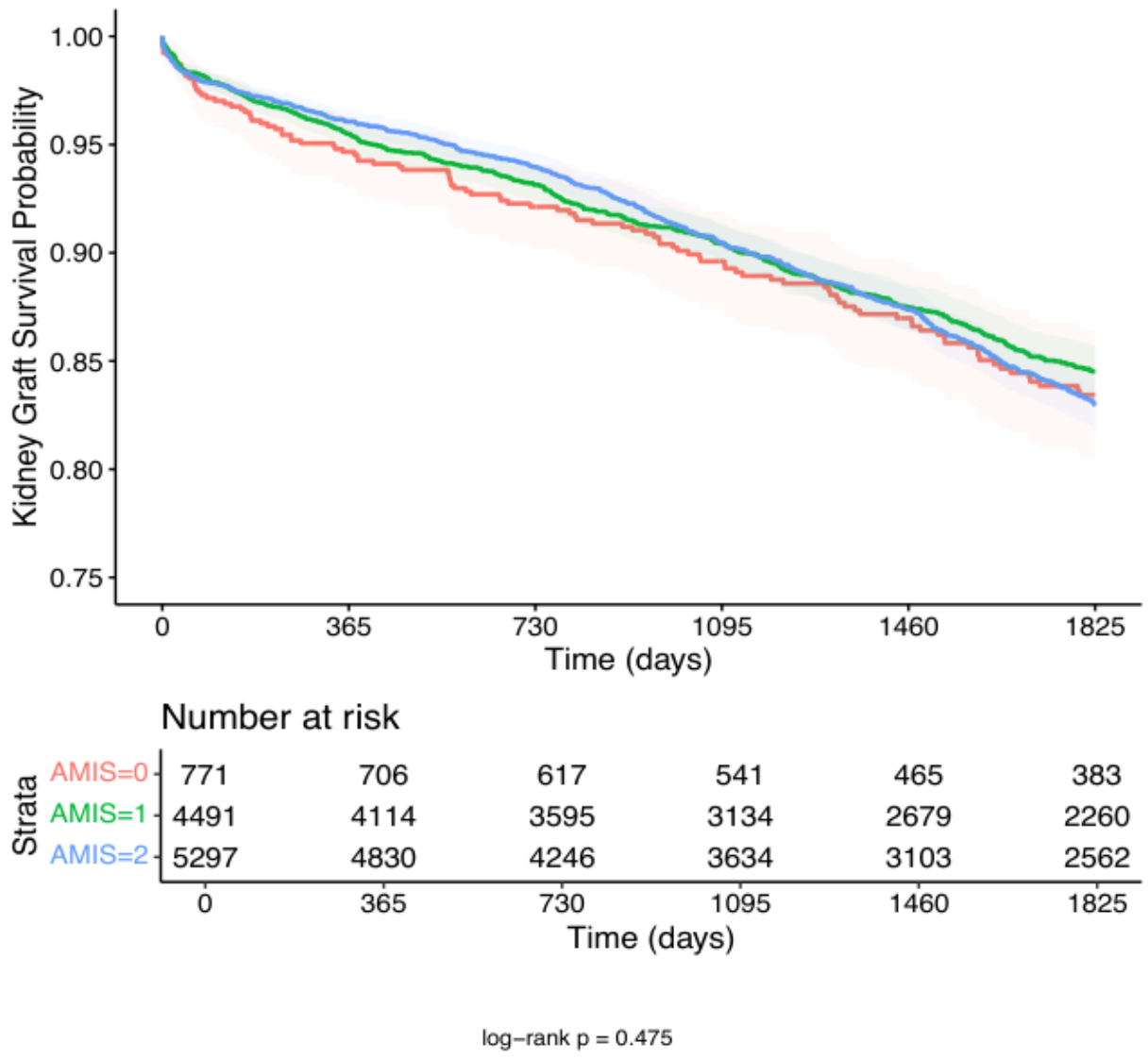

B – HLA B mismatch

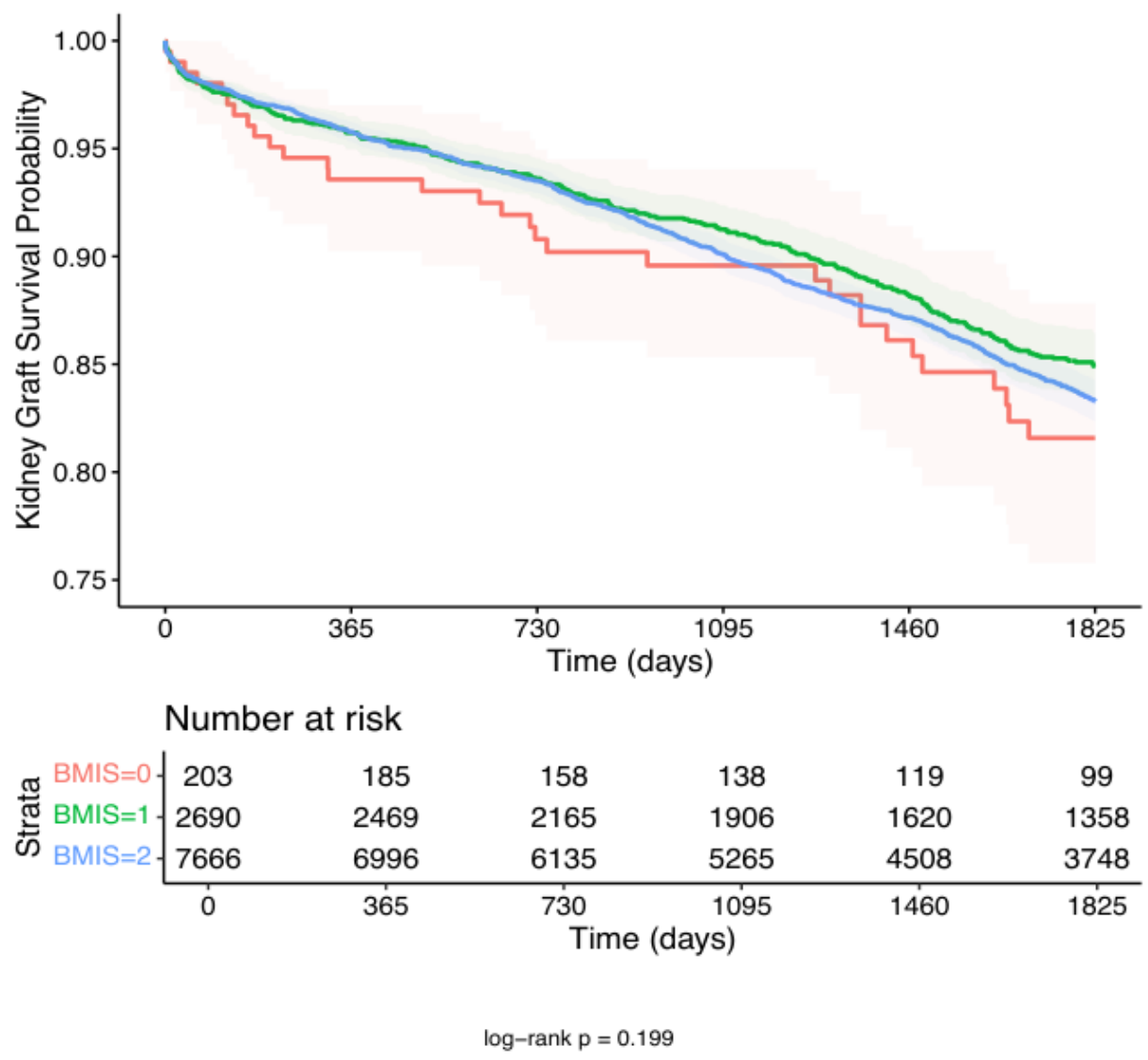

C – HLA Overall mismatch

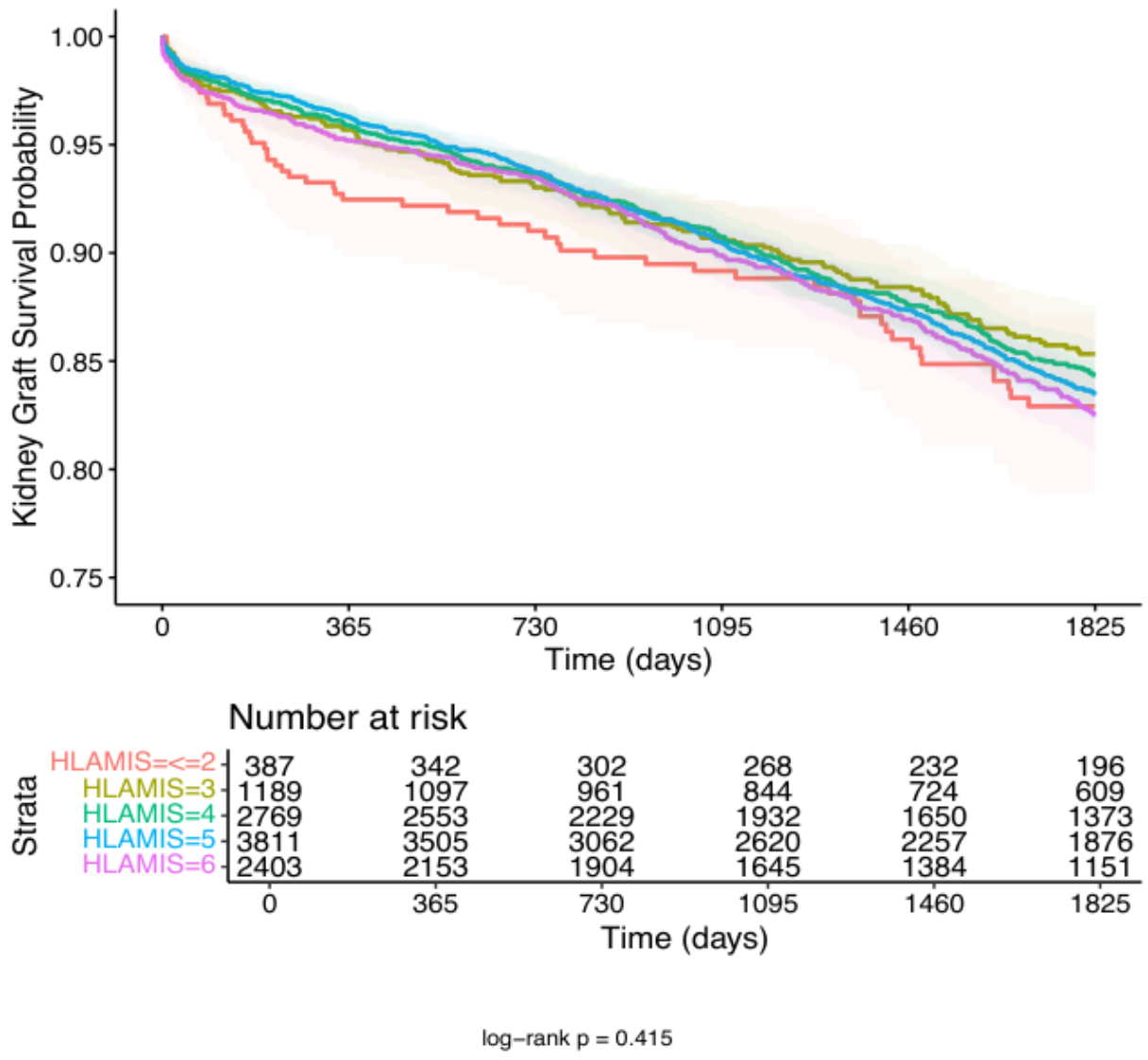

Supplementary Figure 7: Restricted cubic spline model for kidney acute rejection. Restricted cubic spline plots showing associations of donor, recipient, and transplant characteristics with the risk of kidney acute rejection at 1 year, adjusted for all covariates in Supplementary Table 3.

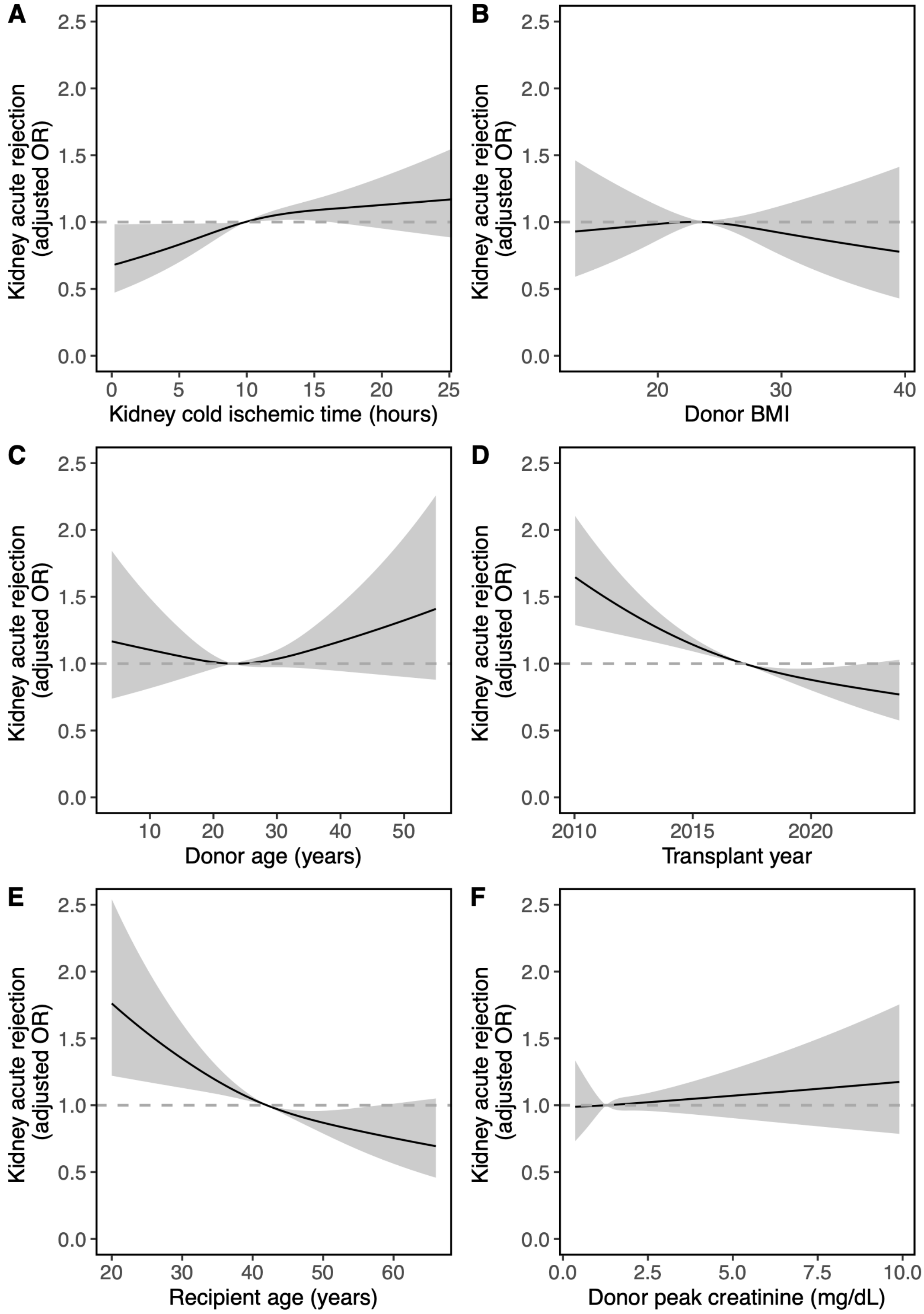

Supplementary Figure 8: Restricted cubic spline model for kidney allograft function. Restricted cubic spline plots showing associations of donor, recipient, and transplant characteristics with estimated glomerular filtration rate (eGFR) at 1 year, adjusted for all covariates in Supplementary Table 4.

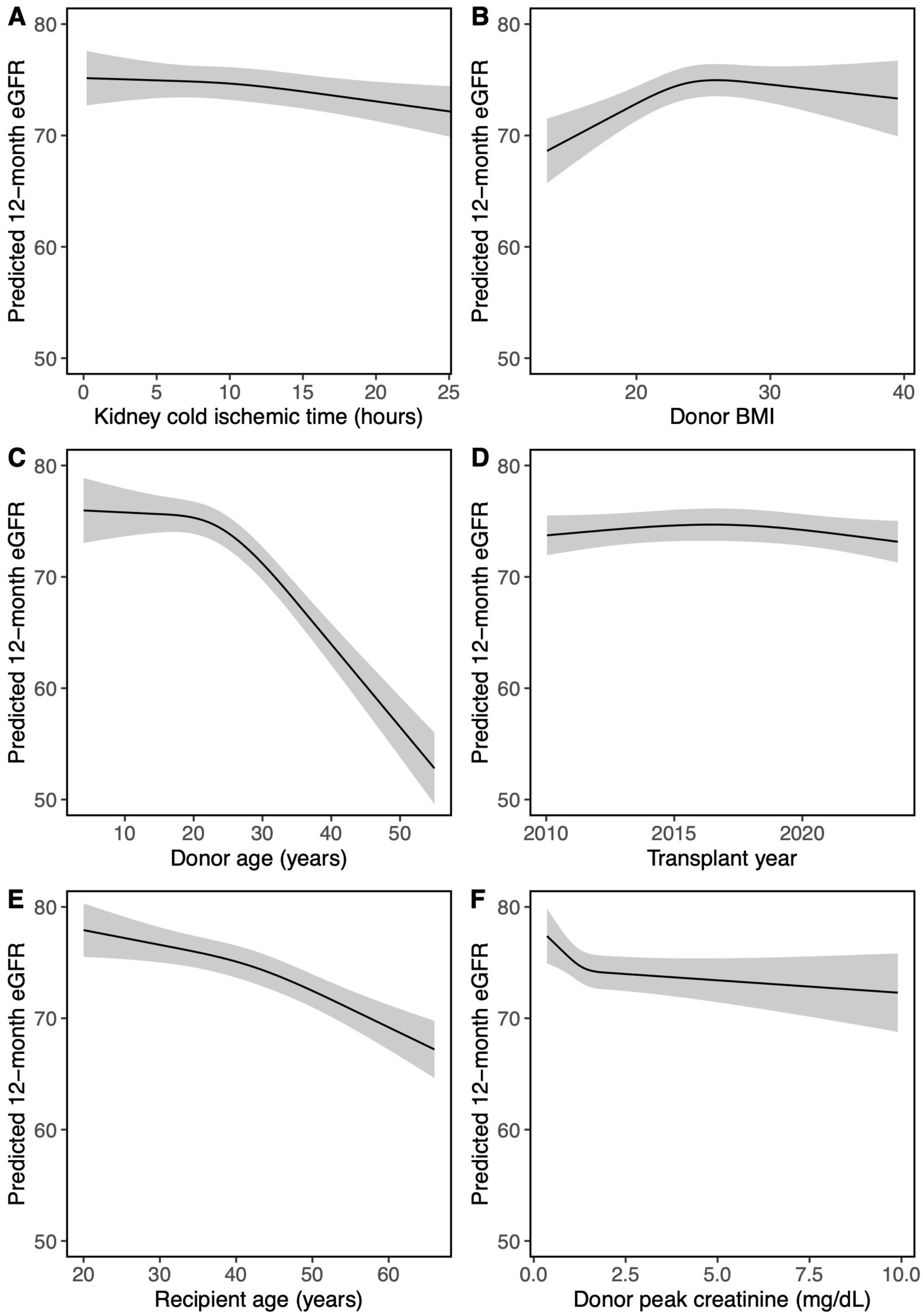

Supplementary Figure 9: Restricted cubic spline model for kidney delayed graft function. Restricted cubic spline plots showing associations of donor, recipient, and transplant characteristics with the risk of kidney delayed graft function (DGF), adjusted for all covariates in Supplementary Table 5.

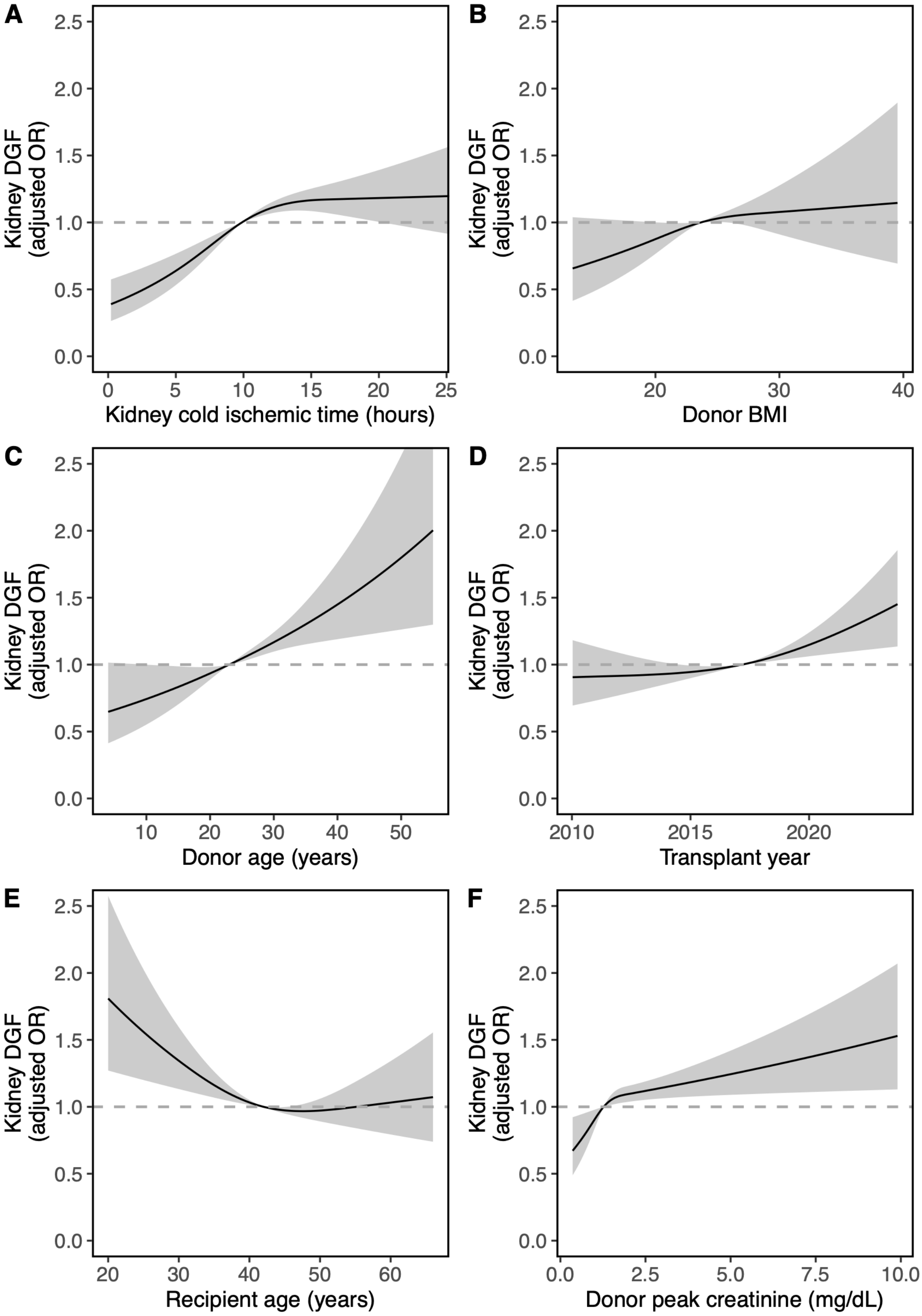

Supplementary Tables

Supplementary Table 1: Detailed donor, recipient, and transplant characteristics. Comprehensive baseline demographics and clinical features of SPK transplant recipients according to the number of HLA-DR mismatches. This table provides an extended version of the summary data shown in Table 1. Abbreviations: SPK, simultaneous pancreas–kidney; BMI, body mass index; ATG, antithymocyte globulin; MMF/MPA, mycophenolate mofetil; CVA, cerebrovascular accident.

Characteristics	0 (N=606)	1 (N=4245)	2 (N=5708)	Overall (N=10559)
**HLA-A mismatches**				
0	72 (11.9%)	334 (7.9%)	365 (6.4%)	771 (7.3%)
1	261 (43.1%)	1896 (44.7%)	2334 (40.9%)	4491 (42.5%)
2	273 (45.0%)	2015 (47.5%)	3009 (52.7%)	5297 (50.2%)
**HLA-B mismatches**				
0	62 (10.2%)	98 (2.3%)	43 (0.8%)	203 (1.9%)
1	197 (32.5%)	1232 (29.0%)	1261 (22.1%)	2690 (25.5%)
2	347 (57.3%)	2915 (68.7%)	4404 (77.2%)	7666 (72.6%)
**Total HLA Mismatches**				
≤2	197 (32.5%)	186 (4.4%)	4 (0.1%)	387 (3.7%)
3	223 (36.8%)	832 (19.6%)	134 (2.3%)	1189 (11.3%)
4	186 (30.7%)	1757 (41.4%)	826 (14.5%)	2769 (26.2%)
5	0 (0%)	1470 (34.6%)	2341 (41.0%)	3811 (36.1%)
6	0 (0%)	0 (0%)	2403 (42.1%)	2403 (22.8%)
**Donor Characteristics**				
**Age**				
Median	23.0 [19.0,29.0]	23.0 [19.0,30.0]	23.0 [18.0,30.0]	23.0 [19.0,30.0]
**Sex**				
Female	188 (31.0%)	1331 (31.4%)	1645 (28.8%)	3164 (30.0%)
Male	418 (69.0%)	2914 (68.6%)	4063 (71.2%)	7395 (70.0%)
**BMI**				
Median	23.6 [21.2,26.3]	23.7 [21.2,26.3]	23.7 [21.2,26.4]	23.7 [21.2,26.3]
Missing	1 (0.2%)	5 (0.1%)	2 (0.0%)	8 (0.1%)
**Ethnicity**				
White, Non-Hispanic	426 (70.3%)	2702 (63.7%)	3256 (57.0%)	6384 (60.5%)
Black, Non-Hispanic	59 (9.7%)	690 (16.3%)	1347 (23.6%)	2096 (19.9%)
Hispanic/Latino	109 (18.0%)	724 (17.1%)	901 (15.8%)	1734 (16.4%)
Asian, Non-Hispanic	6 (1.0%)	82 (1.9%)	133 (2.3%)	221 (2.1%)
Other	6 (1.0%)	47 (1.1%)	71 (1.2%)	124 (1.2%)
**Length of stay**				
Median [Q1,Q3]	4.00 [3.00,6.00]	4.00 [3.00,6.00]	4.00 [3.00,6.00]	4.00 [3.00,6.00]
Missing	3 (0.5%)	14 (0.3%)	19 (0.3%)	36 (0.3%)
**Cause of death**				
Anoxia	137 (22.6%)	841 (19.8%)	1110 (19.4%)	2088 (19.8%)
CVA/Stroke	59 (9.7%)	454 (10.7%)	592 (10.4%)	1105 (10.5%)
Drug Overdose	69 (11.4%)	481 (11.3%)	619 (10.8%)	1169 (11.1%)
Head Trauma	328 (54.1%)	2381 (56.1%)	3269 (57.3%)	5978 (56.6%)

Other	13 (2.1%)	88 (2.1%)	118 (2.1%)	219 (2.1%)
**DCD donor**				
Yes	24 (4.0%)	135 (3.2%)	158 (2.8%)	317 (3.0%)
No	582 (96.0%)	4110 (96.8%)	5550 (97.2%)	10242 (97.0%)
**Insulin Use**				
Yes	333 (55.0%)	2161 (50.9%)	2857 (50.1%)	5351 (50.7%)
No	273 (45.0%)	2081 (49.0%)	2847 (49.9%)	5201 (49.3%)
Missing	0 (0%)	3 (0.1%)	4 (0.1%)	7 (0.1%)
**Smoker**				
Yes	23 (3.8%)	183 (4.3%)	236 (4.1%)	442 (4.2%)
No	577 (95.2%)	4009 (94.4%)	5405 (94.7%)	9991 (94.6%)
Missing	6 (1.0%)	53 (1.2%)	67 (1.2%)	126 (1.2%)
**Heavy Alcohol Use**				
Yes	44 (7.3%)	380 (9.0%)	476 (8.3%)	900 (8.5%)
No	550 (90.8%)	3776 (89.0%)	5108 (89.5%)	9434 (89.3%)
Missing	12 (2.0%)	89 (2.1%)	124 (2.2%)	225 (2.1%)
**Hypertension**				
Yes	23 (3.8%)	186 (4.4%)	221 (3.9%)	430 (4.1%)
No	581 (95.9%)	4046 (95.3%)	5456 (95.6%)	10083 (95.5%)
Missing	2 (0.3%)	13 (0.3%)	31 (0.5%)	46 (0.4%)
**IV Drug Use**				
Yes	44 (7.3%)	353 (8.3%)	402 (7.0%)	799 (7.6%)
No	557 (91.9%)	3849 (90.7%)	5253 (92.0%)	9659 (91.5%)
Missing	5 (0.8%)	43 (1.0%)	53 (0.9%)	101 (1.0%)
**Serum Sodium**				
Median [Q1,Q3]	147 [142,153]	148 [142,153]	148 [142,153]	148 [142,153]
Missing	0 (0%)	0 (0%)	1 (0.0%)	1 (0.0%)
**Blood Urea Nitrogen**				
Median [Q1,Q3]	15.0 [10.3,20.0]	15.0 [10.0,21.0]	15.0 [10.0,21.0]	15.0 [10.0,21.0]
Missing	0 (0%)	0 (0%)	1 (0.0%)	1 (0.0%)
**Peak HbA1c**				
Median [Q1,Q3]	5.30 [5.05,5.50]	5.30 [5.00,5.50]	5.30 [5.10,5.50]	5.30 [5.10,5.50]
Missing	211 (34.8%)	1345 (31.7%)	1821 (31.9%)	3377 (32.0%)
**Transplant Characteristics**				
**Pancreas Preservation Time**				
Median [Q1,Q3]	10.0 [7.59,13.5]	9.90 [7.48,13.0]	9.98 [7.50,13.0]	9.95 [7.50,13.0]
Missing	17 (2.8%)	118 (2.8%)	172 (3.0%)	307 (2.9%)
**Kidney Cold Ischaemic Time**				
Median [Q1,Q3]	10.0 [7.50,13.8]	9.85 [7.29,13.2]	9.88 [7.30,13.0]	9.88 [7.30,13.1]
Missing	9 (1.5%)	76 (1.8%)	100 (1.8%)	185 (1.8%)

**Pancreas Preservation Solution**				
UW	454 (74.9%)	3128 (73.7%)	4234 (74.2%)	7816 (74.0%)
HTK	46 (7.6%)	409 (9.6%)	532 (9.3%)	987 (9.3%)
Other	102 (16.8%)	696 (16.4%)	916 (16.0%)	1714 (16.2%)
Missing	4 (0.7%)	12 (0.3%)	26 (0.5%)	42 (0.4%)
**Duct Management**				
Enteric drainage	551 (90.9%)	3857 (90.9%)	5204 (91.2%)	9612 (91.0%)
Bladder drainage	32 (5.3%)	228 (5.4%)	293 (5.1%)	553 (5.2%)
Other	23 (3.8%)	160 (3.8%)	211 (3.7%)	394 (3.7%)
**Vascular Management**				
Systemic	502 (82.8%)	3560 (83.9%)	4756 (83.3%)	8818 (83.5%)
Portal	102 (16.8%)	667 (15.7%)	921 (16.1%)	1690 (16.0%)
Missing	2 (0.3%)	18 (0.4%)	31 (0.5%)	51 (0.5%)
**Laboratory Values**				
**Peak Lipase**				
Median [Q1,Q3]	41.0 [18.0,91.0]	37.0 [18.0,90.0]	38.0 [18.0,93.0]	38.0 [18.0,92.0]
Missing	3 (0.5%)	15 (0.4%)	32 (0.6%)	50 (0.5%)
**Terminal Lipase**				
Median [Q1,Q3]	30.0 [14.0,69.0]	26.0 [13.0,62.0]	27.0 [13.0,64.0]	26.0 [13.0,63.0]
Missing	3 (0.5%)	13 (0.3%)	30 (0.5%)	46 (0.4%)
**Peak Amylase**				
Median [Q1,Q3]	87.0 [47.0,167]	82.0 [43.5,167]	85.0 [47.0,169]	84.0 [46.0,168]
Missing	0 (0%)	10 (0.2%)	11 (0.2%)	21 (0.2%)
**Terminal Amylase**				
Median [Q1,Q3]	61.0 [35.0,114]	59.0 [33.0,111]	60.5 [35.0,116]	60.0 [34.0,114]
Missing	0 (0%)	6 (0.1%)	6 (0.1%)	12 (0.1%)
**Peak SGOT**				
Median [Q1,Q3]	108 [55.3,239]	102 [57.0,224]	102 [56.0,230]	103 [56.0,229]
Missing	0 (0%)	2 (0.0%)	2 (0.0%)	4 (0.0%)
**SGOT (AST) (u/L)**				
Median [Q1,Q3]	42.0 [26.0,71.0]	42.0 [26.0,78.0]	43.0 [26.0,77.5]	42.0 [26.0,77.0]
Missing	0 (0%)	0 (0%)	1 (0.0%)	1 (0.0%)
**Peak SGPT**				
Median [Q1,Q3]	64.0 [31.3,174]	65.0 [31.0,164]	62.0 [31.0,163]	63.0 [31.0,164]
Missing	0 (0%)	0 (0%)	1 (0.0%)	1 (0.0%)
**SGPT (AST) (u/L)**				
Median [Q1,Q3]	37.0 [22.0,66.0]	36.0 [22.0,69.0]	35.0 [21.0,67.0]	35.0 [22.0,68.0]
Missing	0 (0%)	0 (0%)	1 (0.0%)	1 (0.0%)
**Peak Creatinine (mg/dL)**				

Median [Q1,Q3]	1.27 [1.00,1.54]	1.24 [1.00,1.57]	1.28 [1.00,1.59]	1.27 [1.00,1.58]
**Terminal Creatinine (mg/dL)**				
Median [Q1,Q3]	0.810 [0.650,1.09]	0.850 [0.660,1.10]	0.890 [0.700,1.10]	0.870 [0.680,1.10]
**Recipient Characteristics**				
**Age**				
Median [Q1,Q3]	41.0 [35.0,48.0]	42.0 [35.0,49.0]	41.0 [35.0,49.0]	42.0 [35.0,49.0]
**Sex**				
Female	281 (46.4%)	1629 (38.4%)	2143 (37.5%)	4053 (38.4%)
Male	325 (53.6%)	2616 (61.6%)	3565 (62.5%)	6506 (61.6%)
**BMI**				
Median [Q1,Q3]	25.1 [22.5,27.8]	25.3 [22.7,28.3]	25.3 [22.9,28.1]	25.3 [22.8,28.2]
Missing	0 (0%)	0 (0%)	2 (0.0%)	2 (0.0%)
**Ethnicity**				
White, Non-Hispanic	372 (61.4%)	2344 (55.2%)	2932 (51.4%)	5648 (53.5%)
Black, Non-Hispanic	132 (21.8%)	1083 (25.5%)	1604 (28.1%)	2819 (26.7%)
Hispanic/Latino	88 (14.5%)	622 (14.7%)	894 (15.7%)	1604 (15.2%)
Asian, Non-Hispanic	7 (1.2%)	123 (2.9%)	202 (3.5%)	332 (3.1%)
Other	7 (1.2%)	73 (1.7%)	76 (1.3%)	156 (1.5%)
**CPRA Category**				
≤20	413 (68.2%)	3354 (79.0%)	4656 (81.6%)	8423 (79.8%)
>20	178 (29.4%)	767 (18.1%)	857 (15.0%)	1802 (17.1%)
Missing	15 (2.5%)	124 (2.9%)	195 (3.4%)	334 (3.2%)
**Diabetes Type**				
Type I	532 (87.8%)	3511 (82.7%)	4564 (80.0%)	8607 (81.5%)
Type II	69 (11.4%)	709 (16.7%)	1096 (19.2%)	1874 (17.7%)
Missing	5 (0.8%)	25 (0.6%)	48 (0.8%)	78 (0.7%)
**Dialysis**				
Yes	446 (73.6%)	3176 (74.8%)	4331 (75.9%)	7953 (75.3%)
No	160 (26.4%)	1069 (25.2%)	1377 (24.1%)	2606 (24.7%)
**Waiting Time**				
Median [Q1,Q3]	210 [68.3,493]	200 [68.0,467]	191 [62.0,457]	196 [64.0,463]
**Transplant Year**				
Median [Q1,Q3]	2020 [2010,2020]	2020 [2010,2020]	2020 [2010,2020]	2020 [2010,2020]
**Immunosuppression**				
**Induction Therapy**				
ATG	408 (67.3%)	2846 (67.0%)	3769 (66.0%)	7023 (66.5%)
ATG + Basiliximab	18 (3.0%)	154 (3.6%)	205 (3.6%)	377 (3.6%)
Basiliximab without ATG	39 (6.4%)	232 (5.5%)	280 (4.9%)	551 (5.2%)
Alemtuzumab	87 (14.4%)	633 (14.9%)	852 (14.9%)	1572 (14.9%)
Other	54 (8.9%)	380 (9.0%)	602 (10.5%)	1036 (9.8%)
**Steroid Induction**				

Yes	391 (64.5%)	2735 (64.4%)	3608 (63.2%)	6734 (63.8%)
No	185 (30.5%)	1295 (30.5%)	1807 (31.7%)	3287 (31.1%)
Missing	30 (5.0%)	215 (5.1%)	293 (5.1%)	538 (5.1%)
**Steroid Maintenance**				
Yes	417 (68.8%)	2939 (69.2%)	4050 (71.0%)	7406 (70.1%)
No	159 (26.2%)	1091 (25.7%)	1365 (23.9%)	2615 (24.8%)
Missing	30 (5.0%)	215 (5.1%)	293 (5.1%)	538 (5.1%)
**Tacrolimus+MMF/MPA maintenance**				
Yes	576 (95.0%)	3979 (93.7%)	5342 (93.6%)	9897 (93.7%)
No	17 (2.8%)	140 (3.3%)	202 (3.5%)	359 (3.4%)
Missing	13 (2.1%)	126 (3.0%)	164 (2.9%)	303 (2.9%)
**CMV Matching**				
Positive/Negative: yes	167 (27.6%)	1117 (26.3%)	1492 (26.1%)	2776 (26.3%)
Positive/Negative: no	429 (70.8%)	3052 (71.9%)	4135 (72.4%)	7616 (72.1%)
Missing	10 (1.7%)	76 (1.8%)	81 (1.4%)	167 (1.6%)
**Outcomes**				
**1-year eGFR**				
Median [Q1,Q3]	69.9 [56.3,87.4]	71.5 [56.4,87.1]	70.0 [55.7,85.9]	70.5 [56.0,86.5]
Missing	10 (1.7%)	103 (2.4%)	131 (2.3%)	244 (2.3%)
**1-year Pancreas Rejection**				
Yes	33 (5.4%)	261 (6.1%)	377 (6.6%)	671 (6.4%)
No	439 (72.4%)	2926 (68.9%)	3911 (68.5%)	7276 (68.9%)
Missing	134 (22.1%)	1058 (24.9%)	1420 (24.9%)	2612 (24.7%)
**1-year Kidney Rejection**				
Yes	42 (6.9%)	269 (6.3%)	394 (6.9%)	705 (6.7%)
No	448 (73.9%)	3102 (73.1%)	4108 (72.0%)	7658 (72.5%)
Missing	116 (19.1%)	874 (20.6%)	1206 (21.1%)	2196 (20.8%)

Supplementary Table 2: Multivariable model for pancreas acute rejection. Logistic regression model assessing the association between donor, recipient, and transplant characteristics and the risk of pancreas acute rejection at 1 year, adjusted for HLA mismatches and clinical covariates. Restricted cubic spline terms were included for pancreas preservation time, donor BMI, donor age, treatment year, and recipient age; spline plots are shown in Supplementary Figure 4. Abbreviations: ATG, antithymocyte globulin; MMF/MPA, mycophenolate mofetil; CVA, cerebrovascular accident; RCS, restricted cubic spline.

Variable	Odds ratio (95% CI)	P value
**HLA-DR mismatches**		
0	Ref	-
1	1.186 (0.847 to 1.660)	0.321
2	1.246 (0.899 to 1.728)	0.186
**HLA-A mismatches**		
0	Ref	-
1	0.876 (0.649 to 1.184)	0.390
2	0.978 (0.725 to 1.321)	0.886
**HLA-B mismatches**		
0	Ref	-
1	1.241 (0.677 to 2.274)	0.484
2	1.123 (0.606 to 2.082)	0.713
**Final Flush Solution**		
HTK	0.974 (0.749 to 1.267)	0.844
Other	0.860 (0.686 to 1.078)	0.190
**Induction Therapy**		
ATG	Ref	-
ATG + Basiliximab	0.867 (0.563 to 1.335)	0.516
Basiliximab without ATG	2.031 (1.460 to 2.824)	<0.001
Alemtuzumab	1.716 (1.409 to 2.090)	<0.001
Other	1.030 (0.778 to 1.363)	0.837
**Steroids induction: yes**	1.217 (1.011 to 1.464)	0.038
**Tacrolimus + MMF/MPA maintenance: no**	1.049 (0.697 to 1.579)	0.818
**Donor cause of death**		
Anoxia	Ref	-
CVA/stroke	1.044 (0.799 to 1.363)	0.753
Drug Overdose	0.884 (0.667 to 1.173)	0.393
Head Trauma	0.959 (0.798 to 1.153)	0.659
Other	0.716 (0.400 to 1.282)	0.261
**DCD donor**	1.319 (0.820 to 2.123)	0.253
**Recipient BMI (per unit)**	1.025 (1.006 to 1.044)	0.009
**Recipient diabetes: Type 2**	0.978 (0.788 to 1.214)	0.840
**On dialysis: yes**	1.055 (0.893 to 1.246)	0.530
**CPRA ≤ 20**	Ref	-
**CPRA > 20**	1.131 (0.912 to 1.403)	0.262
**RCS: Pancreas preservation time**	RCS terms	0.211
**RCS: BMI of donor**	RCS terms	0.689
**RCS: Age of donor**	RCS terms	0.039
**RCS: Treatment year**	RCS terms	<0.001

**RCS: Age of recipient**	RCS terms	0.300

Supplementary Table 3: Multivariable model for kidney acute rejection. Logistic regression model assessing the association between donor, recipient, and transplant characteristics and the risk of kidney acute rejection at 1 year, adjusted for HLA mismatches and clinical covariates. Restricted cubic spline terms were included for cold ischaemic time, donor BMI, donor age, treatment year, recipient age, and donor peak creatinine; spline plots are shown in Supplementary Figure 7. Abbreviations: ATG, antithymocyte globulin; MMF/MPA, mycophenolate mofetil; CVA, cerebrovascular accident; RCS, restricted cubic spline.

Variable	Odds Ratio (95% CI)	P value
**HLA-A mismatches**		
0	Ref	-
1	0.923 (0.695 to 1.226)	0.581
2	0.955 (0.716 to 1.275)	0.756
**HLA-B mismatches**		
0	Ref	-
1	1.555 (0.786 to 3.073)	0.204
2	1.356 (0.691 to 2.664)	0.376
**HLA-DR mismatches**		
0	Ref	-
1	0.853 (0.621 to 1.172)	0.326
2	0.979 (0.715 to 1.341)	0.897
**Induction Therapy**		
ATG	Ref	-
ATG + Basiliximab	0.823 (0.534 to 1.268)	0.377
Basiliximab without ATG	1.374 (0.964 to 1.958)	0.079
Alemtuzumab	1.274 (1.046 to 1.551)	0.016
Other	1.256 (0.987 to 1.599)	0.064
**Steroids induction: yes**	1.114 (0.935 to 1.329)	0.227
**Tacrolimus + MMF/MPA maintenance: no**	1.451 (1.034 to 2.037)	0.032
**Donor cause of death**		
Anoxia	Ref	-
CVA/stroke	1.112 (0.837 to 1.476)	0.464
Drug Overdose	1.210 (0.893 to 1.638)	0.219
Head Trauma	1.156 (0.938 to 1.425)	0.173
Other	1.253 (0.749 to 2.096)	0.391
**DCD donor**	1.112 (0.683 to 1.811)	0.670
**Recipient BMI (per unit)**	1.036 (1.017 to 1.056)	<0.001
**Recipient diabetes: Type 2**	1.009 (0.807 to 1.262)	0.936
**On dialysis: yes**	1.196 (0.999 to 1.432)	0.051
**CPRA ≤ 20**	Ref	-
**CPRA > 20**	1.046 (0.844 to 1.297)	0.680
**RCS: Kidney Cold Ischaemic Time**	RCS terms	0.026
**RCS: BMI of donor**	RCS terms	0.709
**RCS: Age of donor**	RCS terms	0.359
**RCS: Treatment year**	RCS terms	<0.001
**RCS: Age of recipient**	RCS terms	<0.001

**RCS: Donor peak creatinine**	RCS terms	0.729

Supplementary Table 4: Multivariable model for kidney allograft function. Linear regression model evaluating the association between donor, recipient, and transplant characteristics and estimated glomerular filtration rate (eGFR) at 1 year, adjusted for HLA mismatches and clinical covariates. Restricted cubic spline terms were included for cold ischaemic time, donor BMI, donor age, treatment year, recipient age, and donor peak creatinine; spline plots are shown in Supplementary Figure 8. Abbreviations: ATG, antithymocyte globulin; MMF/MPA, mycophenolate mofetil; CVA, cerebrovascular accident; RCS, restricted cubic spline; eGFR, estimated glomerular filtration rate.

Variable	Hazard ratio (95% CI)	P value
**HLA-A mismatches**		
0	Ref	-
1	0.437 (-1.411 to 2.284)	0.643
2	-0.323 (-2.155 to 1.510)	0.730
**HLA-B mismatches**		
0	Ref	-
1	1.641 (-1.820 to 5.103)	0.353
2	1.019 (-2.387 to 4.425)	0.558
**HLA-DR mismatches**		
0	Ref	-
1	0.637 (-1.425 to 2.699)	0.545
2	-0.511 (-2.560 to 1.539)	0.625
**Induction Therapy**		
ATG	Ref	-
ATG + Basiliximab	5.795 (3.280 to 8.310)	<0.001
Basiliximab without ATG	0.212 (-1.882 to 2.305)	0.843
Alemtuzumab	-0.647 (-1.970 to 0.675)	0.337
Other	-2.701 (-4.375 to -1.028)	0.002
**Steroids induction: yes**	-1.334 (-2.372 to -0.296)	0.012
**Tacrolimus + MMF/MPA maintenance: no**	-6.693 (-9.394 to -3.991)	<0.001
**Donor cause of death**		
Anoxia	Ref	-
CVA/stroke	-1.372 (-3.214 to 0.470)	0.144
Drug Overdose	0.266 (-1.498 to 2.030)	0.768
Head Trauma	2.010 (0.785 to 3.234)	0.001
Other	-0.765 (-4.125 to 2.595)	0.655
**DCD donor**	-2.705 (-5.428 to 0.019)	0.052
**Recipient BMI (per unit)**	-0.707 (-0.828 to -0.586)	<0.001
**Recipient diabetes: Type 2**	-0.100 (-1.403 to 1.202)	0.880
**On dialysis: yes**	2.030 (0.946 to 3.114)	<0.001
**CPRA ≤ 20**	Ref	-
**CPRA > 20**	-2.334 (-3.574 to -1.094)	<0.001
**RCS: Kidney Cold Ischaemic Time**	RCS terms	0.019
**RCS: BMI of donor**	RCS terms	<0.001
**RCS: Age of donor**	RCS terms	<0.001
**RCS: Treatment year**	RCS terms	0.219

**RCS: Age of recipient**	RCS terms	<0.001
**RCS: Donor peak creatinine**	RCS terms	0.009

Supplementary Table 5: Multivariable model for kidney delayed graft function. Logistic regression model assessing the association between donor, recipient, and transplant characteristics and the risk of kidney delayed graft function (DGF), adjusted for HLA mismatches and clinical covariates. Restricted cubic spline terms were included for cold ischaemic time, donor BMI, donor age, treatment year, recipient age and donor peak creatinine; spline plots are shown in Supplementary Figure 9

Variable	Odds Ratio (95% CI)	P value
**HLA-A mismatches**		
0	Ref	-
1	0.976 (0.733 to 1.300)	0.869
2	1.095 (0.825 to 1.452)	0.530
**HLA-B mismatches**		
0	Ref	-
1	1.032 (0.606 to 1.757)	0.908
2	0.926 (0.548 to 1.564)	0.773
**HLA-DR mismatches**		
0	Ref	-
1	0.951 (0.691 to 1.308)	0.758
2	1.074 (0.784 to 1.471)	0.657
**Induction Therapy**		
ATG	Ref	-
ATG + Basiliximab	0.718 (0.468 to 1.102)	0.130
Basiliximab without ATG	1.160 (0.845 to 1.591)	0.359
Alemtuzumab	1.035 (0.847 to 1.266)	0.734
Other	1.256 (0.993 to 1.589)	0.058
**Steroids induction: yes**	0.854 (0.731 to 0.998)	0.048
**Tacrolimus + MMF/MPA maintenance: no**	1.836 (1.320 to 2.552)	<0.001
**Donor cause of death**		
Anoxia	Ref	-
CVA/stroke	1.043 (0.800 to 1.360)	0.755
Drug Overdose	0.840 (0.651 to 1.084)	0.180
Head Trauma	0.848 (0.708 to 1.016)	0.073
Other	0.841 (0.502 to 1.408)	0.510
**DCD donor**	3.777 (2.851 to 5.003)	<0.001
**Recipient BMI (per unit)**	1.043 (1.025 to 1.062)	<0.001
**Recipient diabetes: Type 2**	1.183 (0.984 to 1.422)	0.073
**On dialysis: yes**	2.228 (1.819 to 2.728)	<0.001
**CPRA ≤ 20**	Ref	-
**CPRA > 20**	0.915 (0.755 to 1.109)	0.367
**RCS: Kidney Cold Ischaemic Time**	RCS terms	<0.001
**RCS: BMI of donor**	RCS terms	0.060
**RCS: Age of donor**	RCS terms	<0.001
**RCS: Treatment year**	RCS terms	<0.001
**RCS: Age of recipient**	RCS terms	0.002
**RCS: Donor peak creatinine**	RCS terms	0.001
